# Copper-catalyzed aminooxygenation of styrenes with *N*-fluorobenzenesulfonimide and *N*-hydroxyphthalimide derivatives

**DOI:** 10.3762/bjoc.11.293

**Published:** 2015-12-24

**Authors:** Yan Li, Xue Zhou, Guangfan Zheng, Qian Zhang

**Affiliations:** 1 Department of Chemistry, Northeast Normal University, Changchun 130024, China

**Keywords:** aminooxygenation of styrenes, copper catalysts, *N*-fluorobenzenesulfonimide, *N*-hydroxyphthalimide derivatives

## Abstract

A copper-catalyzed aminooxygenation reaction of styrenes with *N*-fluorobenzenesulfonimide and *N*-hydroxyphthalimide derivatives has been developed. The aminooxygenation product could be converted into the corresponding alcohol or free amine through the cleavage of the N–O or C–N bond of the *N*-hydroxyphthalimide moiety.

## Findings

**Figure 1:**
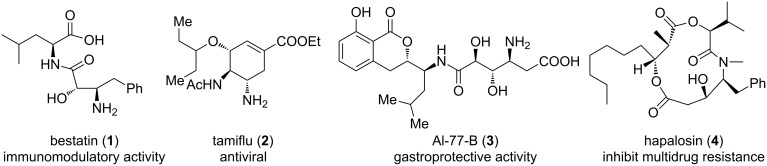
Bioactive compounds containing 1,2-aminoalcohol motif.

Direct aminooxygenation of alkenes provides a straightforward and powerful approach to construct the 1,2-aminoalcohol skeleton [1], which is ubiquitous in bioactive compounds (such as the drugs bestatin (**1**) and tamiflu (**2**), the natural products Al-77-B (**3**) and hapolosin (**4**); Figure 1) [2] and has also been widely used as chiral ligands and auxiliaries in asymmetric synthesis [3]. Therefore, the development of a new aminooxygenation reaction is still highly attractive [4]. Most of the existing aminooxygenation reactions involve an intramolecular cyclization step [5–33] to provide various valuable cyclic compounds. Comparatively, methods for an intermolecular three-component aminooxygenation reaction are considerably less established. In 2006, Stahl and co-workers reported a Pd-catalyzed aminooxygenation reaction of alkenes with phthalimide and (diacetoxyiodo)benzene through *cis*-aminopalladation and S_N_2 C–O bond formation [34]. In 2013, Zhu and co-workers described an *n*-Bu_4_NI-catalyzed aminooxygenation of inactive alkenes with benzotriazole and water which underwent a nitrogen-centred radical addition and a nucleophilic oxygen attack [35]. Very recently, Studer and co-workers presented an aminooxygenation of alkenes with *N*-fluorobenzenesulfonimide (NFSI) and sodium 2,2,6,6-tetramethylpiperidine-1-olate (TEMPONa) via nitrogen-centred radical addition to the alkene followed by trapping of 2,2,6,6-tetramethylpiperidine-*N*-oxyl (TEMPO) [36].

**Scheme 1:**
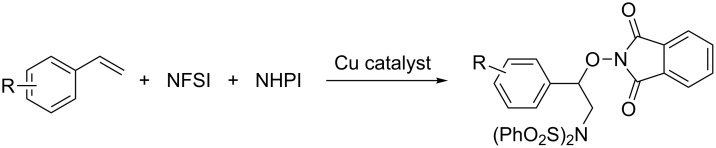
Copper-catalyzed radical aminooxygenation reaction of styrenes.

NFSI is a very interesting reagent. Besides classic electrophilic fluorination reagent [37], it has been used not only as fluoride-atom transfer reagent [38–40] but also as nucleophilic/radical amination reagent [41]. We are highly interested in the multiple reaction modes of NFSI [37–41], especially as a nitrogen-centred radical. In this context, we have realized copper-catalyzed benzylic sp^3^ C–H amination [42], aminative multiple functionalization of alkynes [43], diamination, aminocyanation [44] and aminofluorination of alkenes [45], as well as amination of allenes [46]. Encouraged by these results, we try to develop copper-catalyzed aminooxygenation of alkenes by using NFSI. Herein, we report a simple and efficient copper-catalyzed three-component aminooxygenation reaction of styrenes with NFSI and *N*-hydroxyphthalimide (NHPI) derivatives (Scheme 1).

Initially, we conducted the three-component amnooxygenation of styrene **1a** with NFSI and NHPI (**2a**). After the reaction of **1a** (0.3 mmol), NFSI (0.3 mmol, 1.0 equiv) and **2a** (0.45 mmol, 1.5 equiv) was performed in the presence of Cu(OTf)_2_ (10 mol %) in dichloromethane (DCM, 2 mL) under nitrogen atmosphere at 70 °C for 10.0 h, the desired aminooxygenation product **3a** was obtained in 39% yield (Table 1, entry 1). A variety of copper salts such as CuCl, CuBr, CuI, [(CH_3_CN)_4_Cu]PF_6_, CuCN, Cu(acac)_2_, Cu(OAc)_2_, CuBr_2_ and CuCl_2_ were examined (Table 1, entries 2–10). We found that CuCl_2_ was the most effective catalyst, affording **3a** in 55% yield (Table 1, entry 10). No reaction was observed in the absence of copper salts (Table 1, entry 11). Next, the reaction solvents were scanned. 1,2-Dichloroethane (DCE) and CH_3_CN were not efficient solvents, providing **3a** in 9% and 20% yields, respectively (Table 1, entries 12 and 13). Using CHCl_3_ as the solvent, only a trace amount of **3a** was observed (Table 1, entry 14). No reaction occurred in the solvents DMF, DMSO and THF (Table 1, entries 15–17). A relatively lower temperature (45 °C) only afforded a trace amount of **3a** (Table 1, entry 18). Increasing the temperature to 90 °C or 110 °C, **3a** was obtained in 45% and 40% yields, respectively (Table 1, entries 19 and 20). The ratio of substrates distinctly influenced the reaction (Table 1, entries 21–23). Changing the ratio from 1:1:1.5 (**1a**:NFSI:**2a**) to 1:2:2 or 1:2:3 (**2a**:NFSI:**1a**) led to much better yields (Table 1, entries 21 and 22). To our delight, when the ratio was 1:4:3 (**2a**:NFSI:**1a**), **3a** was obtained in 76% yield (Table 1, entry 23).

**Table 1:** The optimization of reaction conditions^a^.

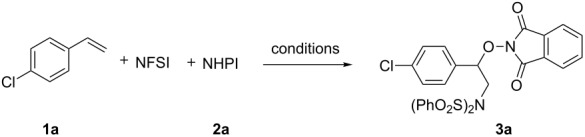				

Entry^a^	Catalyst	Solvent	Temp (°C)	Yield^b^ (%)

1	Cu(OTf)_2_	DCM	70	39
2	CuCl	DCM	70	48
3	CuBr	DCM	70	43
4	CuI	DCM	70	30
5	[(CH_3_CN)_4_Cu]PF_6_	DCM	70	50
6	CuCN	DCM	70	16
7	Cu(acac)_2_	DCM	70	48
8	Cu(OAc)_2_	DCM	70	51
9	CuBr_2_	DCM	70	54
10	CuCl_2_	DCM	70	55
11	none	DCM	70	NR^c^
12	CuCl_2_	DCE	70	9
13	CuCl_2_	CH_3_CN	70	20
14	CuCl_2_	CHCl_3_	70	trace
15	CuCl_2_	DMF	70	NR^c^
16	CuCl_2_	DMSO	70	NR^c^
17	CuCl_2_	THF	70	NR^c^
18	CuCl_2_	DCM	45	trace
19	CuCl_2_	DCM	90	45
20	CuCl_2_	DCM	110	40
21^d^	CuCl_2_	DCM	70	70
22^e^	CuCl_2_	DCM	70	73
**23^f^**	**CuCl_2_**	**DCM**	**70**	**76**

^a^Reaction conditions: **1a** (0.3 mmol), NFSI (0.3 mmol, 1.0 equiv), **2a** (0.45 mmol, 1.5 equiv), catalyst (10 mol %), solvent (2.0 mL), N_2_, 10.0 h. ^b^Isolated yields. ^c^NR: no reaction. ^d^**1a**:NFSI:**2a** = 2.0:2.0:1.0 ^e^**1a**:NFSI:**2a** = 3.0:2.0:1.0. ^f^**1a**:NFSI:**2a** = 3.0:4.0:1.0.

**Figure 2:**
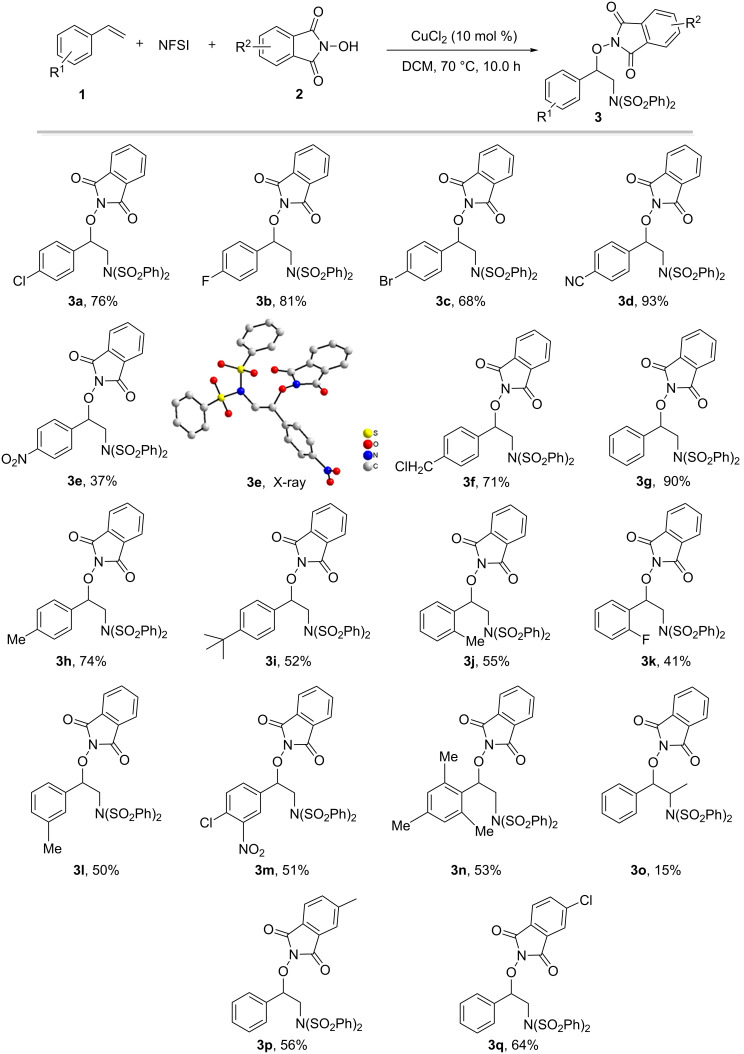
The copper-catalyzed three-component aminooxygenation of styrenes with NFSI and NHPI derivatives. Reaction conditions: **1** (0.9 mmol, 3.0 equiv), NFSI (1.2 mmol, 4.0 equiv), **2** (0.3 mmol, 1.0 equiv), CuCl_2_ (10 mol %), DCM (2.0 mL), N_2_, 70 °C, 10.0 h. Isolated yields.

With the optimized reaction conditions in hand (Table 1, entry 23), the scope of this copper-catalyzed aminooxygenation reaction was examined (Figure 2). Styrenes with electron-withdrawing (**1a**–**f**) or electron-donating (**1h** and **1i**) groups were viable, providing the corresponding 1,2-aminoalcohol derivatives in good yields. It is worth noting that functionalities such as F, Cl, Br, CN, and NO_2_ groups, which could easily undergo further transformations, were intact after the reaction (**3a**–**e**). The structure of **3e** was confirmed by X-ray crystallographic analysis [47]. The substituent at the *ortho* (**3j** and **3k**) or *meta* (**3l**) position of the aromatic ring did not hinder the reaction (41–55% yields). Similarly, for disubstituted (**1m**) and trisubstituted (**1n**) substrates, the aminooxygenation underwent smoothly, providing the corresponding products **3m** (51%) and **3n** (53%). The *trans*-β-methylstyrene (**1o**) afforded the desired product **3o** in a low yield (15%). In addition, NHPI derivatives **2b** and **2c** were suitable nitrogen sources and the desired **3p** and **3q** were obtained in 56% and 64%, respectively. For 4-methoxystyrene (**1r**), no aminooxygenation reaction occurred.

**Scheme 2:**
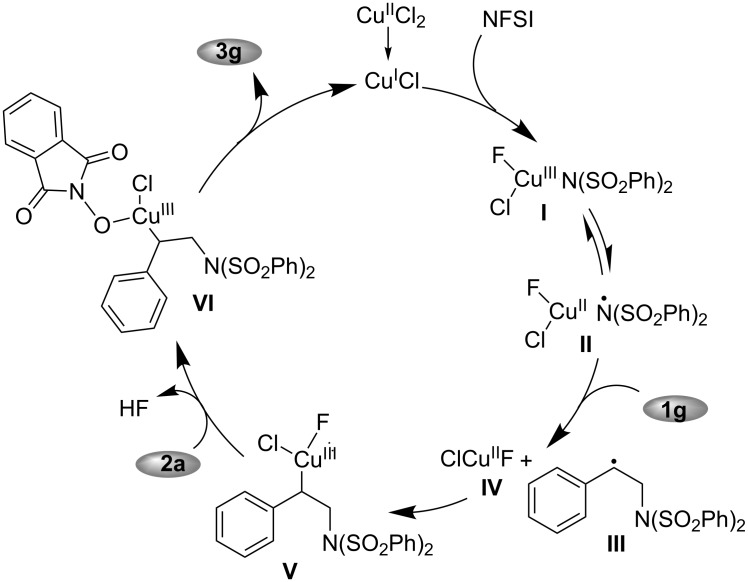
The plausible mechanism.

Based on these experimental results and our previous investigations [42–46,48], a plausible mechanism for the copper-catalyzed three-component aminooxygenation of styrenes with NFSI an NHPI is shown in Scheme 2. Initially, the oxidation of Cu(I) with NFSI provided F–Cu(III)–N complex **I**, which could transform into a copper(II)-stabilized benzenesulfonimide radical **II** through a redox isomerization equilibrium. Next, the intermolecular radical addition of **II** to styrene **1g** took place, producing benzylic radical **III** and Cu(II)–F species **IV**. The combination of the intermediates **III** and **IV** gave the Cu(III) species **V** having a C–Cu bond, which reacted with **2a** to generate Cu(III)–O species **VI**, along with the loss of HF. Finally, the reductive elimination of **VI** afforded aminooxygenation product **3g**.

**Scheme 3:**
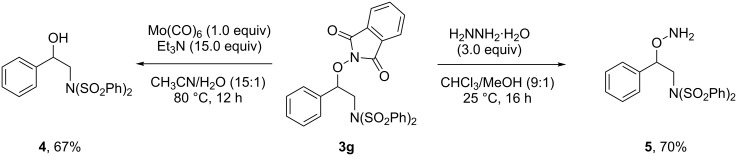
Selective reduction of the aminooxygenation product.

Finally, we tried to investigate the synthetic value of our new aminooxygenation method. Then, the selective reduction of **3g** was conducted (Scheme 3). The cleavage of the N–O bond in **3g** readily occurred with Mo(CO)_6_/Et_3_N at 80 °C to give alcohol **4** [36] in 67% yield. Treatment of **3g** with NH_2_NH_2_·H_2_O under mild conditions (25 °C) in CHCl_3_/MeOH gave free amine **5** in 70% yield.

In summary, we have developed a novel copper-catalyzed three-component aminooxygenation reaction of styrenes with NFSI and NHPI derivatives. Furthermore, the aminooxygenation product could be easily converted into the corresponding alcohol or free amine through the cleavage of the N–O or C–N bond of the NHPI moiety. Further studies are underway in our lab.

## Supporting Information

File 1 Experimental part.
