# Supplementary material for: Copper-catalyzed aminooxygenation of styrenes with N-fluorobenzenesulfonimide and N-hydroxyphthalimide derivatives
Source: Beilstein J Org Chem. 2015 Dec 24;11:2721–6. doi: 10.3762/bjoc.11.293 (PMC4734344; doi:10.3762/bjoc.11.293)
Supplement: File 1 — Experimental part. [file Beilstein_J_Org_Chem-11-2721-s001.pdf]

Supporting Information

for

Copper-catalyzed aminooxygenation of styrenes with *N*-fluorobenzenesulfonimide and *N*-hydroxyphthalimide derivatives

Yan Li[1], Xue Zhou[1], Guangfan Zheng[1] and Qian Zhang*[1]

Address: [1]Department of Chemistry, Northeast Normal University, Changchun 130024, China

Email: Qian Zhang* - zhangq651@nenu.edu.cn

* Corresponding author

Experimental part

Table of Contents

I. General information……………………….....………...S2

II. Synthesis procedure..………………………………………………………………..……...S2

III. Analytical data of compounds 3–5……………………………………………..…………..S4

IV. Crystal structure of 3e……………………………………………………………………..S12

V. ^{1}H NMR and ^{13}C NMR spectra copies of compounds 3–5……………………………..…….S13

I. General information

All reagents were purchased from commercial sources and used without further treatment, unless otherwise indicated. All aminooxygenation reactions were run under nitrogen atmosphere. ^{1}H NMR spectra were recorded at 25 ºC on a Varian 500 MHz spectrometer, ^{13}C NMR spectra were recorded at 25 ºC on a Varian 125 MHz or a Bruker 100 MHz spectrometer, and TMS as internal standard. IR spectra (KBr) were recorded on a Magna-560 FTIR spectrophotometer in the range of 400–4000 cm^{-1}. Melting points were obtained with a micro melting point XT4A Beijing Keyi electrooptic apparatus and are uncorrected. High resolution mass spectra were recorded on Bruker microtof. All reactions were monitored by TLC with Taizhou GF$_{254}$ silica gel coated plates. Flash column chromatography was carried out using 300–400 mesh silica gel at increased pressure.

II. Synthesis procedure

i. General procedure for the synthesis of 3 (3a as an example).

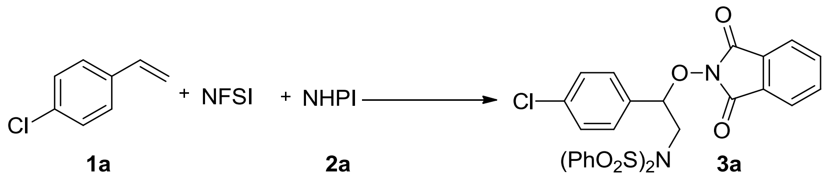

In a nitrgeon-filled glove-box, *N*-hydroxyphthalimide (NHPI, **2a**, 48.9 mg, 0.3 mmol), styrene **1a** (114.7 μL, 0.9 mmol), *N*-fluorobenzenesulfonimide (NFSI, 378.4 mg, 1.2 mmol), $CuCl_2$ (4.0 mg, 0.03 mmol), dichloromethane (DCM, 2.0 mL) and were combine in a 10 mL vial sealed with a Teflon-lined cap. The reaction mixture was stirred at 70 ^{o}C for 10 h. After the reaction quenched by water, the mixture was extracted with CH_2Cl_2 (3 × 5.0 mL). The combined organic layers were dried with Na_2SO_4 and evaporated in-vacuo. The residue was purified by column chromatography on silica gel with gradient of petroleum ether/ethyl acetate (8:1) afforded the product **3a** (136.13 mg, 76%) as a white solid.

ii. Selective reduction of aminooxygenation 3g.

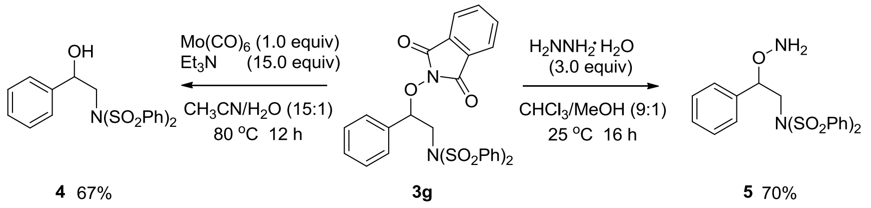

For **4**:

To a 5 mL mixed solvent of CH_3CN/H_2O (15:1, v/v) in a 10 mL round-bottom flask, *N*-(2-((1,3-dioxoisoindolin-2-yl)oxy)-2-phenylethyl)-*N*-(phenylsulfonyl)benzenesulfonamide (**3g**, 0.3 mmol, 168.8 mg), $Mo(CO)_6$ (1.0 equiv, 0.3 mmol, 79.2 mg) and Et_3N (15.0 equiv, 4.5 mmol, 0.65 mL) were added. The reaction mixture was heated at 80 °C for 12 hours. After the reaction quenched by

water, the mixture was extracted with CH_2Cl_2 (3 × 5.0 mL). The combined organic layers were dried with Na_2SO_4 and evaporated in-vacuo. The residue was purified by column chromatography on silica gel with gradient of petroleum ether/ethyl acetate (4:1) afforded the product **4** (83.9 mg, 67%) as yellow liquid.

For **5**:

To a 5 mL mixed solvent of CH_3Cl/MeOH (9:1, v/v) in a 10 mL round-bottom flask, *N*-(2-((1,3-dioxoisoindolin-2-yl)oxy)-2-phenylethyl)-*N*-(phenylsulfonyl)benzenesulfonamide (**3g**, 0.3 mmol, 168.8 mg) and $NH_2NH_2{\bullet}H_2O$ (3.0 equiv, 0.9 mmol, 0.043 mL) were added. The reaction mixture was stirred at 25 °C for 16 hours. After the reaction quenched by water, the mixture was extracted with CH_2Cl_2 (3 × 5.0 mL). The combined organic layers were dried with Na_2SO_4 and evaporated in-vacuo. The residue was purified by column chromatography on silica gel with gradient of petroleum ether/ethyl acetate (3:1) afforded the product **5** (90.8 mg, 70%) as yellow liquid.

III. Analytical data of compounds 3–5

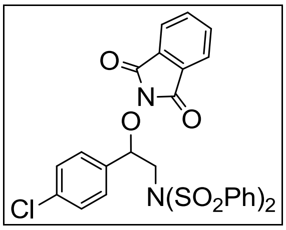

N-(2-(4-Chlorophenyl)-2-((1,3-dioxoisoindolin-2-yl)oxy)ethyl)-N-(phenylsulfonyl)benzenesulfon-amide (3a)

76% Yield. White solid. mp: 259-251 °C; **^{1}H NMR** (500 MHz, $CDCl_3$): δ = 4.03 (dd, J_1 = 5.0 Hz, J_2 = 16.0 Hz, 1H), 4.37 (dd, J_1 = 8.0 Hz, J_2 = 16.0 Hz, 1H), 5.77 (dd, J_1 = 5.0 Hz, J_2 = 8.0 Hz, 1H), 7.31 (d, J = 8.5 Hz, 2H), 7.45-7.48 (m, 6H), 7.53-7.56 (m, 2H), 7.69-7.72 (m, 4H), 8.01-8.02 (m, 4H). **^{13}C NMR** (125 MHz, $CDCl_3$): δ = 51.8, 87.2, 123.5, 128.6, 128.7, 128.8, 128.9, 129.6, 133.6, 133.8, 134.5, 135.6, 139.1, 162.9. **HRMS** (ESI-TOF) Calcd for $C_{28}H_{21}ClN_2O_7S_2Na$, $[M+Na]^+$ 619.0371; Found 619.0373.

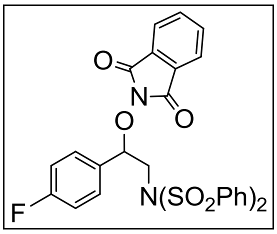

N-(2-((1,3-Dioxoisoindolin-2-yl)oxy)-2-(4-fluorophenyl)ethyl)-N-(phenylsulfonyl)benzenesulfon-amide (3b)

81% Yield. White solid. mp: 150-152 °C; **^{1}H NMR** (500 MHz, $CDCl_3$): δ = 4.02 (dd, J_1 = 4.5 Hz, J_2 = 16.0 Hz, 1H), 4.40 (dd, J_1 = 8.0 Hz, J_2 = 16.0 Hz, 1H), 5.77 (dd, J_1 = 4.5 Hz, J_2 = 8.0 Hz, 1H), 7.03 (t, J = 8.5 Hz, 2H), 7.47 (t, J = 8.0 Hz, 4H), 7.50-7.56 (m, 4H), 7.69-7.73 (m, 4H), 8.04 (d, J = 7.5 Hz, 4H). **^{13}C NMR** (125 MHz, $CDCl_3$): δ = 51.9, 87.2, 115.7 (d, J = 21.5 Hz), 123.4, 128.6, 128.7, 130.1 (d, J = 8.4 Hz), 130.9 (d, J = 3.1 Hz), 133.7, 134.5, 139.2, 162.9, 163.4 (d, J = 247.4 Hz). **HRMS** (ESI-TOF) Calcd for $C_{28}H_{21}FN_2NaO_7S_2$, $[M+Na]^+$ 603.0666; Found 603.0673.

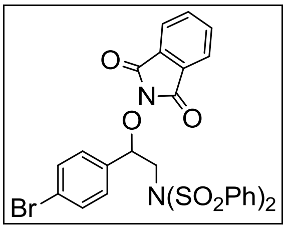

N-(2-(4-Bromophenyl)-2-((1,3-dioxoisoindolin-2-yl)oxy)ethyl)-N-(phenylsulfonyl)benzenesulfon-amide (3c)

68% Yield. White solid. mp: 177-179 °C; **^{1}H NMR** (500 MHz, $CDCl_3$): δ = 4.03 (dd, J_1 = 5.0 Hz, J_2 = 16.5

Hz, 1H), 4.36 (dd, J_1 = 8.0 Hz, J_2 = 16.5 Hz, 1H), 5.76 (dd, J_1 = 5.0 Hz, J_2 = 7.5 Hz, 1H), 7.42 (d, J = 8.5 Hz, 2H), 7.46-7.49 (m, 6H), 7.55 (t, J = 7.0 Hz, 2H), 7.69-7.72 (m, 4H), 8.01 (d, J = 8.5, 4H). **^{13}C NMR** (125 MHz, $CDCl_3$): δ = 51.8, 87.3, 123.5, 123.9, 128.5, 128.7, 128.8, 129.8, 131.8, 133.8, 134.1, 134.5, 139.1, 162.9. **HRMS** (ESI-TOF) Calcd for $C_{28}H_{21}BrN_2NaO_7S_2$, $[M+Na]^+$ 662.9866; Found 662.9880.

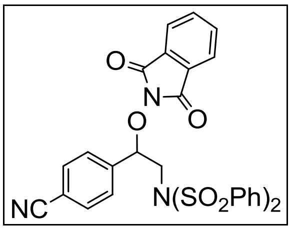

***N*-(2-(4-Cyanophenyl)-2-((1,3-dioxoisoindolin-2-yl)oxy)ethyl)-*N*-(phenylsulfonyl)benzenesulfonamide (3d)**

93% Yield. White solid. mp: 280-281 °C; **^{1}H NMR** (500 MHz, $CDCl_3$): δ = 4.00 (dd, J_1 = 4.5 Hz, J_2 = 16.0 Hz, 1H), 4.40 (dd, J_1 = 8.0 Hz, J_2 = 16.0 Hz, 1H), 5.87(dd, J_1 = 5.0 Hz, J_2 = 8.0 Hz, 1H), 7.49 (t, J = 8.0 Hz, 4H), 7.57 (t, J = 7.5 Hz, 2H), 7.66 (d, J = 8.5, 2H), 7.70 (d, J = 8.5, 2H), 7.74 (m, 4H), 8.05 (d, J = 7.5 Hz, 4H). **^{13}C NMR** (125 MHz, $CDCl_3$): δ = 51.9, 87.2, 113.4, 118.3, 123.6, 128.4, 128.5, 128.6, 128.7, 128.8, 132.4, 132.7, 133.9, 134.3, 134.7, 139.0, 140.4, 162.8. **HRMS** (ESI-TOF) Calcd for $C_{29}H_{21}N_3NaO_7S_2$, $[M+Na]^+$ 610.0713; Found 610.0725.

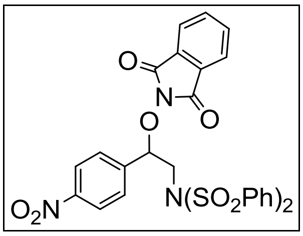

***N*-(2-((1,3-Dioxoisoindolin-2-yl)oxy)-2-(4-nitrophenyl)ethyl)-*N*-(phenylsulfonyl)benzenesulfonamide (3e)**

37% Yield. White solid. mp: 244-246 °C; **^{1}H NMR** (500 MHz, $CDCl_3$): δ = 4.00 (dd, J_1 = 4.5 Hz, J_2 = 16.0 Hz, 1H), 4.42 (dd, J_1 = 8.0 Hz, J_2 = 16.5 Hz, 1H), 5.91 (dd, J_1 = 5.0 Hz, J_2 = 7.5 Hz, 1H), 7.47 (t, J = 8.5 Hz, 4H), 7.56 (t, J = 7.0 Hz, 2H), 7.72-7.76 (m, 6H), 8.05 (d, J = 8.0 Hz, 4H), 8.19 (d, J = 8.0 Hz, 2H). **^{13}C NMR** (125 MHz, $CDCl_3$): δ = 51.9, 87.0, 123.6, 123.7, 128.4, 128.7, 128.8, 128.9, 133.9, 134.7, 139.0, 142.3, 148.6, 162.8. **HRMS** (ESI-TOF) Calcd for $C_{28}H_{21}N_3NaO_9S_2$, $[M+Na]^+$ 630.0611; Found 630.0614.

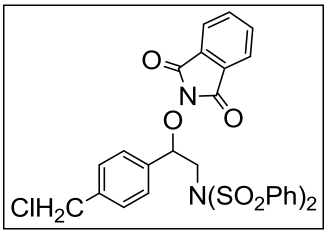

***N*-(2-(4-(Chloromethyl)phenyl)-2-((1,3-dioxoisoindolin-2-yl)oxy)ethyl)-*N*-(phenylsulfonyl)benzene-sulfonamide (3f)**

71% Yield. White solid. mp: 180-182 °C; **^{1}H NMR** (500 MHz, $CDCl_3$): δ = 4.04 (dd, J_1 = 5.0 Hz, J_2 = 16.5 Hz 1H), 4.39 (dd, J_1 = 8.0 Hz, J_2 = 16.5 Hz, 1H), 4.57 (s, 2H), 5.80 (dd, J_1 = 4.5 Hz, J_2 = 8.0 Hz, 1H), 7.40 (d, J = 8.0 Hz, 2H), 7.47 (t, J = 8.0 Hz, 4H), 7.52-7.56 (m, 4H), 7.70-7.71 (m, 4H), 8.03 (d, J = 8.0 Hz, 4H). **^{13}C NMR** (125 MHz, $CDCl_3$): δ = 45.6, 51.8, 87.5, 123.4, 128.4, 128.5, 128.5, 128.6, 128.6, 128.7, 133.7, 134.4, 135.3, 138.8, 139.1, 162.9. **HRMS** (ESI-TOF) Calcd for $C_{29}H_{23}ClN_2O_7S_2$, $[M+H]^+$ 611.0708; Found 611.0702.

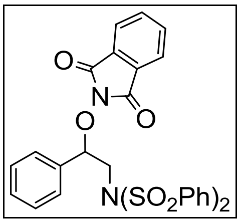

***N*-(2-((1,3-Dioxoisoindolin-2-yl)oxy)-2-phenylethyl)-*N*-(phenylsulfonyl)benzenesulfonamide (3g)**

90% Yield. White solid. mp: 302-303 °C; **^{1}H NMR** (500 MHz, $CDCl_3$): δ = 4.03 (dd, J_1 = 4.0 Hz, J_2 = 16.0 Hz, 1H), 4.43 (dd, J_1 = 8.5 Hz, J_2 = 16.0 Hz, 1H), 5.81 (dd, J_1 = 4.0 Hz, J_2 = 7.5 Hz, 1H), 7.36-7.37 (m, 3H), 7.44-7.54 (m, 4H),7.51-7.54 (m, 4H), 7.67-7.71 (m, 4H), 8.06 (d, J = 8.0 Hz, 4H). **^{13}C NMR** (125 MHz, $CDCl_3$): δ = 52.0, 87.8, 123.3, 128.1, 128.5, 128.6, 128.7, 129.6, 133.6, 134.4, 135.0, 139.2, 162.8. **HRMS** (ESI-TOF) Calcd for $C_{28}H_{22}N_2NaO_7S_2$, $[M+Na]^+$ 585.0761; Found 585.0759.

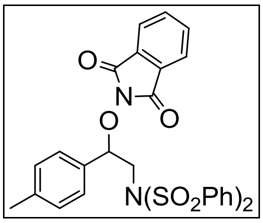

***N*-(2-((1,3-Dioxoisoindolin-2-yl)oxy)-2-(*p*-tolyl)ethyl)-*N*-(phenylsulfonyl)benzenesulfonamide (3h)**

74% Yield. White solid. mp: 202-203 °C; **^{1}H NMR** (500 MHz, $CDCl_3$): δ = 2.34 (s, 3H), 4.01 (dd, J_1 = 4.5 Hz, J_2 = 16.0 Hz, 1H), 4.41 (dd, J_1 = 8.0 Hz, J_2 = 16.0 Hz, 1H), 5.76 (dd, J_1 = 4.5 Hz, J_2 = 8.0 Hz, 1H), 7.16 (d, J = 8.0 Hz, 2H), 7.41-7.46 (m, 6H), 7.53 (t, J = 7.5 Hz, 2H), 7.66-7.71 (m, 4H), 8.04 (d, J = 7.5 Hz, 4H). **^{13}C NMR** (125 MHz, $CDCl_3$): δ = 21.3, 52.0, 87.7, 123.3, 128.1, 128.7, 128.8, 129.3, 131.9, 133.6,

134.3, 139.3, 139.6, 162.9. **HRMS** (ESI-TOF) Calcd for $C_{29}H_{24}N_2NaO_7S_2$, $[M+Na]^+$ 599.0917; Found 599.0922.

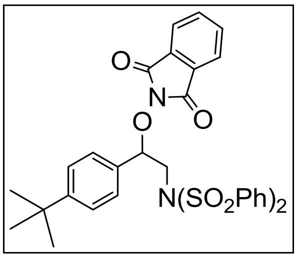

***N*-(2-(4-(*tert*-Butyl)phenyl)-2-((1,3-dioxoisoindolin-2-yl)oxy)ethyl)-*N*-(phenylsulfonyl)benzene-sulfonamide (3i)**

52% Yield. White solid. mp: 156-158 °C; **^{1}H NMR** (400 MHz, $CDCl_3$): δ = 1.30 (s, 9H), 4.05 (dd, J_1 = 4.0 Hz, J_2 = 16.0 Hz 1H), 4.40 (dd, J_1 = 8.4 Hz, J_2 = 16.4 Hz, 1H), 5.79 (dd, J_1 = 4.0 Hz, J_2 = 8.0 Hz, 1H), 7.39-7.46 (m, 10H), 7.69 (m, 4H), 8.04 (d, J = 7.2 Hz, 4H). **^{13}C NMR** (125 MHz, $CDCl_3$): δ = 31.2, 34.6, 52.1, 87.8, 123.3, 125.5, 127.7, 128.6, 128.8, 131.9, 133.6, 134.2, 139.4, 152.6, 162.9. **HRMS** (ESI-TOF) Calcd for $C_{32}H_{30}N_2NaO_7S_2$, $[M+Na]^+$ 641.1387; Found 641.1388.

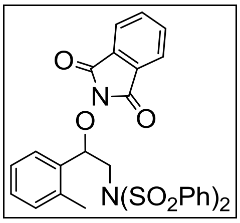

***N*-(2-((1,3-Dioxoisoindolin-2-yl)oxy)-2-(*o*-tolyl)ethyl)-*N*-(phenylsulfonyl)benzenesulfonamide (3j)**

55% Yield. White solid. mp: 246-247 °C; **^{1}H NMR** (500 MHz, $CDCl_3$): δ = 2.35 (s, 3H), 4.00 (dd, J_1 = 3.5 Hz, J_2 = 16.0 Hz, 1H), 4.40 (dd, J_1 = 8.5 Hz, J_2 = 16.0 Hz, 1H), 6.13 (dd, J_1 = 3.5 Hz, J_2 = 8.0 Hz, 1H), 7.10 (d, J = 7.5 Hz, 1H), 7.24 (t, J = 7.5 Hz, 1H), 7.30 (t, J = 7.5 Hz, 1H), 7.44 (t, J = 8.0 Hz, 4H), 7.51 (t, J = 7.5 Hz, 2H), 7.69-7.70 (m, 4H), 7.78 (d, J = 6.0 Hz, 1H), 8.05 (d, J = 7.5 Hz, 4H). **^{13}C NMR** (125 MHz, $CDCl_3$): δ = 19.2, 51.8, 84.5, 123.4, 126.3, 127.9, 128.6, 128.7, 128.8, 129.3, 130.6, 133.4, 133.6, 134.4, 137.3, 139.4, 163.0. **HRMS** (ESI-TOF) Calcd for $C_{29}H_{24}N_2NaO_7S_2$, $[M+Na]^+$ 599.0917; Found 599.0923.

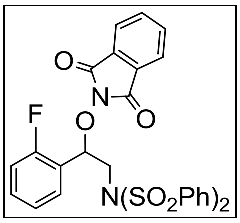

***N*-(2-((1,3-Dioxoisoindolin-2-yl)oxy)-2-(2-fluorophenyl)ethyl)-*N*-(phenylsulfonyl)benzenesulfonamide (3k)**

41% Yield. White solid. mp: 282-284 °C; **^{1}H NMR** (500 MHz, $CDCl_3$): δ = 4.07 (dd, J_1 = 4.0 Hz, J_2 = 16.0 Hz, 1H), 4.57 (dd, J_1 = 8.0 Hz, J_2 = 16.0 Hz, 1H), 6.01 (dd, J_1 = 4.5 Hz, J_2 = 8.0 Hz, 1H), 7.03 (t, J = 9.0 Hz, 1H), 7.19 (t, J = 8.0 Hz, 1H), 7.35-7.36 (m, 1H), 7.45-7.48 (m, 4H), 7.53 (t, J = 7.5 Hz, 2H), 7.70-7.71 (m, 5H), 8.04 (d, J = 7.5 Hz, 4H). **^{13}C NMR** (125 MHz, $CDCl_3$): δ = 50.8, 82.7, 115.7 (d, J = 21.3 Hz), 122.4, 122.5, 123.4, 124.3 (d, J = 3.5 Hz), 128.6, 128.7, 128.8, 129.8, 131.3, 131.4, 133.7, 134.4, 139.2, 161.1 (d, J = 248.6 Hz), 162.8. **HRMS** (ESI-TOF) Calcd for $C_{28}H_{22}FN_2O_7S_2$, $[M+H]^+$ 581.0847; Found 581.0833.

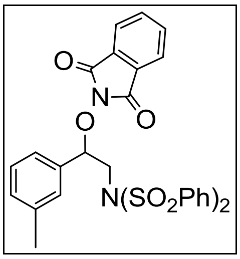

***N*-(2-((1,3-Dioxoisoindolin-2-yl)oxy)-2-(*m*-tolyl)ethyl)-*N*-(phenylsulfonyl)benzenesulfonamide (3l)**

50% Yield. White solid. mp: 196-197 °C; **^{1}H NMR** (500 MHz, $CDCl_3$): δ = 2.33 (s, 3H), 4.03 (dd, J_1 = 4.5 Hz, J_2 = 16.5 Hz, 1H), 4.41 (dd, J_1 = 8.0 Hz, J_2 = 16.5 Hz, 1H), 5.75 (dd, J_1 = 4.5 Hz, J_2 = 8.5 Hz, 1H), 7.17 (d, J = 7.5 Hz, 1H), 7.24-7.27 (m, 1H), 7.31 (s, 1H), 7.35 (d, J = 8.0 Hz, 1H), 7.45 (t, J = 7.5 Hz, 4H), 7.52 (t, J = 7.0 Hz, 2H), 7.67-7.71 (m, 4H), 8.04 (d, J = 7.5 Hz, 4H). **^{13}C NMR** (125 MHz, $CDCl_3$): δ = 21.3, 52.1, 87.9, 123.3, 125.1, 128.5, 128.6, 128.7, 128.8, 128.9, 130.3, 133.6, 134.3, 134.9, 138.3, 139.3, 162.9. **HRMS** (ESI-TOF) Calcd for $C_{29}H_{24}N_2NaO_7S_2$, $[M+Na]^+$ 599.0917; Found 599.0928.

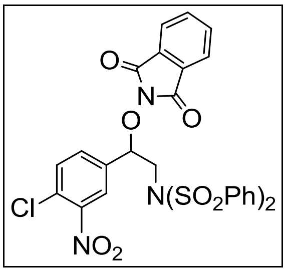

***N*-(2-(4-Chloro-3-nitrophenyl)-2-((1,3-dioxoisoindolin-2-yl)oxy)ethyl)-*N*-(phenylsulfonyl)benzenesulfonamide (3m)**

51% Yield. White solid. mp: 250-252 °C; **^{1}H NMR** (400 MHz, $CDCl_3$): δ = 4.04 (dd, J_1 = 5.6 Hz, J_2 = 16.0

Hz, 1H), 4.39 (dd, J_1 = 7.6 Hz, J_2 = 16.0 Hz, 1H), 5.79 (dd, J_1 = 5.6 Hz, J_2 = 7.6 Hz, 1H), 7.47-7.60 (m, 7H), 7.74-7.79 (m, 5H), 7.95 (d, J = 1.6 Hz, 1H), 8.03 (d, J = 7.6 Hz, 4H). **^{13}C NMR** (125 MHz, $CDCl_3$): δ = 51.6, 86.3, 123.7, 125.2, 128.2, 128.5, 128.7, 128.9, 132.2, 132.5, 134.0, 134.8, 135.8, 138.9, 147.7, 162.8. **HRMS** (ESI-TOF) Calcd for $C_{28}H_{21}ClN_3O_9S_2$, $[M+H]^+$ 642.0402; Found 642.0405.

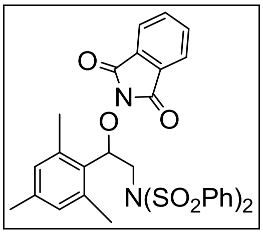

***N*-(2-((1,3-Dioxoisoindolin-2-yl)oxy)-2-mesitylethyl)-*N*-(phenylsulfonyl)benzenesulfonamide (3n)**

53% Yield. White solid. mp: 239-240 °C; **^{1}H NMR** (500 MHz, $CDCl_3$): δ = 2.15 (s, 3H), 2.25 (s, 3H), 2.75 (s, 3H), 4.30 (dd, J_1 = 6.0 Hz, J_2 = 15.5 Hz, 1H), 4.65 (dd, J_1 = 7.0 Hz, J_2 = 16.5 Hz, 1H), 6.09 (t, J = 6.5 Hz, 1H), 6.68 (s, 1H), 6.93 (s, 1H), 7.42 (t, J = 7.5 Hz, 4H), 7.53 (t, J = 7.5 Hz, 2H), 7.69-7.74 (m, 4H), 7.94 (d, J = 8.0 Hz, 4H). **^{13}C NMR** (125 MHz, $CDCl_3$): δ = 20.5, 20.6, 20.9, 49.8, 85.6, 123.4, 128.6, 128.7, 128.7, 129.2, 131.0, 133.6, 134.4, 138.4, 138.8, 139.1, 163.1. **HRMS** (ESI-TOF) Calcd for $C_{31}H_{28}N_2NaO_7S_2$, $[M+Na]^+$ 627.1230; Found 627.1233.

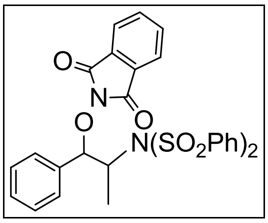

***N*-(1-((1,3-Dioxoisoindolin-2-yl)oxy)-1-phenylpropan-2-yl)-*N*-(phenylsulfonyl)benzenesulfonamide (3o)**

15% Yield. White solid. mp: 140-141 °C; **^{1}H NMR** (500 MHz, $CDCl_3$): δ = 1.74 (d, J = 6.5 Hz, 3H), 4.76-4.79 (m, 1H), 5.84 (d, J = 9.0 Hz, 1H), 7.23 (t, J = 7.5 Hz, 2H), 7.32 (t, J = 6.5 Hz, 3H), 7.38 (d, J = 7.5 Hz, 2H), 7. 50 (t, J = 7.5 Hz, 5H), 7.67 (t, J = 6.0 Hz, 5H), 7.81 (d, J = 7.5 Hz, 2H). **^{13}C NMR** (125 MHz, $CDCl_3$): δ = 18.6, 59.5, 92.0, 123.4, 128.1, 128.3, 128.5, 128.6, 128.9, 129.2, 129.5, 130.6, 133.3, 134.1, 134.3, 163.2. **HRMS** (ESI-TOF) Calcd for $C_{29}H_{24}N_2NaO_7S_2$, $[M+Na]^+$ 599.0917; Found 599.0931.

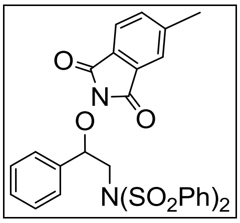

N-(2-((5-Methyl-1,3-dioxoisoindolin-2-yl)oxy)-2-phenylethyl)-N-(phenylsulfonyl)benzenesulfonamide (3p)

56% Yield. White solid. mp: 169-170 °C; **^{1}H NMR** (400 MHz, $CDCl_3$): δ = 2.47 (s, 3H), 4.05 (dd, J_1 = 4.4 Hz, J_2 = 16.0 Hz, 1H), 4.42 (dd, J_1 = 8.0 Hz, J_2 = 16.0 Hz, 1H), 5.80 (dd, J_1 = 4.4 Hz, J_2 = 8.0 Hz, 1H), 7.36-7.38 (m, 3H), 7.44-7.59 (m, 11H), 8.03-8.05 (m, 4H). **^{13}C NMR** (125 MHz, $CDCl_3$): δ = 22.1, 52.0, 87.8, 123.3, 123.9, 125.9, 128.2, 128.6, 128.7, 128.8, 129.6, 133.6, 134.8, 135.1, 139.3, 145.7, 163.0, 163.1. **HRMS** (ESI-TOF) Calcd for $C_{29}H_{24}N_2NaO_7S_2$, $[M+Na]^+$599.0917; Found 599.0961.

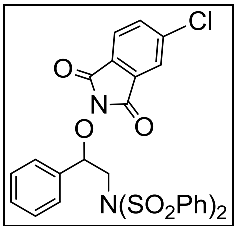

N-(2-((5-Chloro-1,3-dioxoisoindolin-2-yl)oxy)-2-phenylethyl)-N-(phenylsulfonyl)benzenesulfonamide (3q)

64% Yield. White solid. mp: 372-373 °C; **^{1}H NMR** (500 MHz, $CDCl_3$): δ = 4.04 (dd, J_1 = 4.0 Hz, J_2 = 16.5 Hz, 1H), 4.32 (dd, J_1 = 8.5 Hz, J_2 = 16.5 Hz, 1H), 5.78 (dd, J_1 = 4.0 Hz, J_2 = 8.0 Hz, 1H), 7.36-7.37 (m, 3H), 7.45 (d, J = 8.0 Hz, 4H), 7.51-7.54 (m, 4H), 7.64-7.66 (m, 3H), 8.04 (d, J = 8.0 Hz, 4H). **^{13}C NMR** (125 MHz, $CDCl_3$): δ = 52.0, 88.1, 123.8, 124.7, 126.6, 128.2, 128.7, 128.8, 129.7, 130.2, 133.7, 134.4, 134.8, 139.3, 141.1, 161.6, 162.0. **HRMS** (ESI-TOF) Calcd for $C_{28}H_{21}N_2NaO_7S_2$, $[M+Na]^+$619.0371; Found 619.0363.

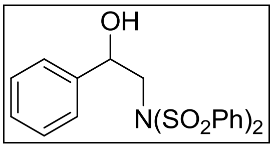

N-(2-Hydroxy-2-phenylethyl)-N-(phenylsulfonyl)benzenesulfonamide (4) [1]

67% Yield. The yield was determined by ^{1}H NMR of the crude reaction mixture with anisole as internal standard. The aryl-containing impurities originated from petrol ether as one component of the eluent, which is difficult to be get rid of. White liquid. **^{1}H NMR** (500 MHz, $CDCl_3$): δ = 2.85 (d, J = 4.5 Hz, 1H), 3.82-3.90 (m, 2H), 5.12-5.15 (m, 1H), 7.31 (t, J = 7.5 Hz, 1H), 7.37 (t, J = 7.5 Hz, 2H), 7.42 (d, J = 7.5 Hz, 2H), 7.56 (t, J = 8.0 Hz, 4H), 7.66 (t, J = 7.5 Hz, 2H), 8.08 (d, J = 8.0 Hz, 4H). **^{13}C NMR**

(125 MHz, $CDCl_3$): δ = 55.6, 72.8, 125.9, 128.2, 128.5, 128.7, 129.1, 134.1, 139.3, 140.7. **HRMS** (ESI-TOF) calcd for $C_{20}H_{19}NNaO_5S_2$, $[M+Na]^+$440.0597; Found: 440.0597.

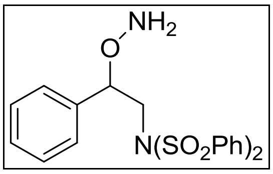

***N*-(2-(Aminooxy)-2-phenylethyl)-*N*-(phenylsulfonyl)benzenesulfonamide (5)**

70% Yield. Yellow liquid. **^{1}H NMR** (400 MHz, $CDCl_3$): δ = 3.65 (dd, J_1 = 2.8 Hz, J_2 = 15.6 Hz, 1H), 4.15 (dd, J_1 = 10.0 Hz, J_2 = 16.0 Hz, 1H), 4.76 (s, 1H), 4.96 (dd, J_1 = 2.4 Hz, J_2 = 9.6 Hz, 1H), 7.33-7.38 (m, 5H), 7.55 (t, J = 8.0 Hz, 4H), 7.64 (dd, J_1 = 1.2 Hz, J_2 = 7.6 Hz, 2H), 8.09-8.12 (m, 4H). **^{13}C NMR** (125 MHz, $CDCl_3$): δ = 52.9, 85.7, 126.8, 128.4, 128.5, 128.8, 128.9, 129.1, 133.8, 138.5, 139.9. HRMS (ESI-TOF) calcd for $C_{20}H_{21}N_2O_5S_2$, $[M+H]^+$433.0886; Found: 433.0883.

Reference

1. Li, Y.; Hartmann, M.; Daniliuc, C. G.; Studer, A. *Chem. Commun*. **2015**, *51*, 5706–5709.

IV. Crystal structure of 3e

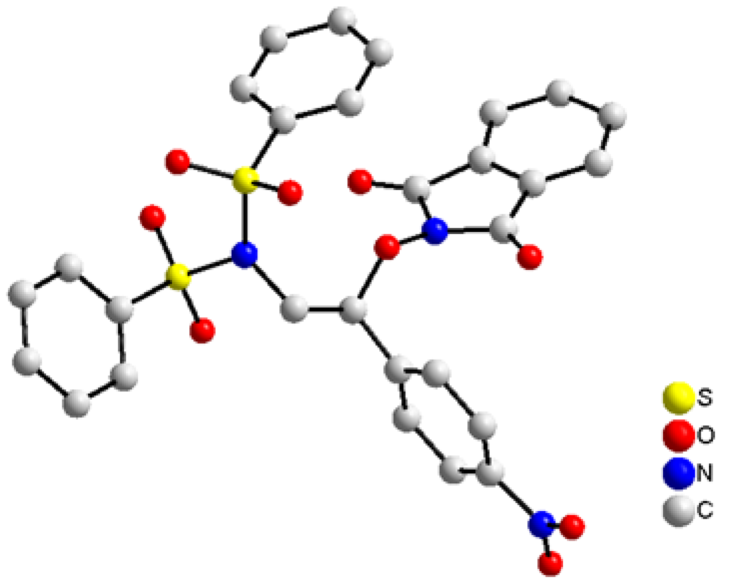

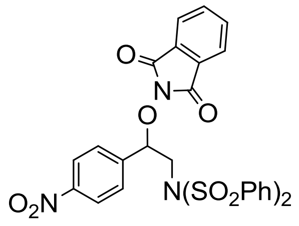

3e

Table 1. Crystal data and structure refinement for **3e**

CCDC number	1422654
Empirical formula	$C_{28} H_{21} N_3 O_9 S_2$
Formula weight	607.60
Temperature	293(2) K
Wavelength	0.71073 A
Crystal system, space group	Triclinic, P-1
Unit cell dimensions	a = 7.695(5) A alpha = 93.793(5) deg.<br>b = 13.108(5) A beta = 100.632(5) deg.<br>c = 13.514(5) A gamma = 96.966(5) deg.
Volume	1324.3(11) A^3
Z, Calculated density	2, 1.524 Mg/m^3
Reflections collected / unique	7207 / 5117 [R(int) = 0.0262]
F(000)	628
Absorption correction	Semi-empirical from equivalents
Theta range for data collection	1.54 to 26.04 deg.
Refinement method	Full-matrix least-squares on F^2
Data / restraints / parameters	5117 / 0 / 379
Goodness-of-fit on F^2	1.042
Final R indices [I>2sigma(I)]	R_1 = 0.0615, wR_2 = 0.1614
R indices (all data)	R_1 = 0.0908, wR_2 = 0.2058
Largest diff. peak and hole	0.552 and -0.495 e.A^-3
$^aR_1 = \Sigma||F_o|-|Fc||/\Sigma|Fo|$; $^bwR_2 = \Sigma[w(F_o^2-F_c^2)^2]/\Sigma[w(F_o^2)^2]^{1/2}$	

V. ^{1}H NMR and ^{13}C NMR spectra copies of compounds 3–5

^{1}H MNR of **3a**

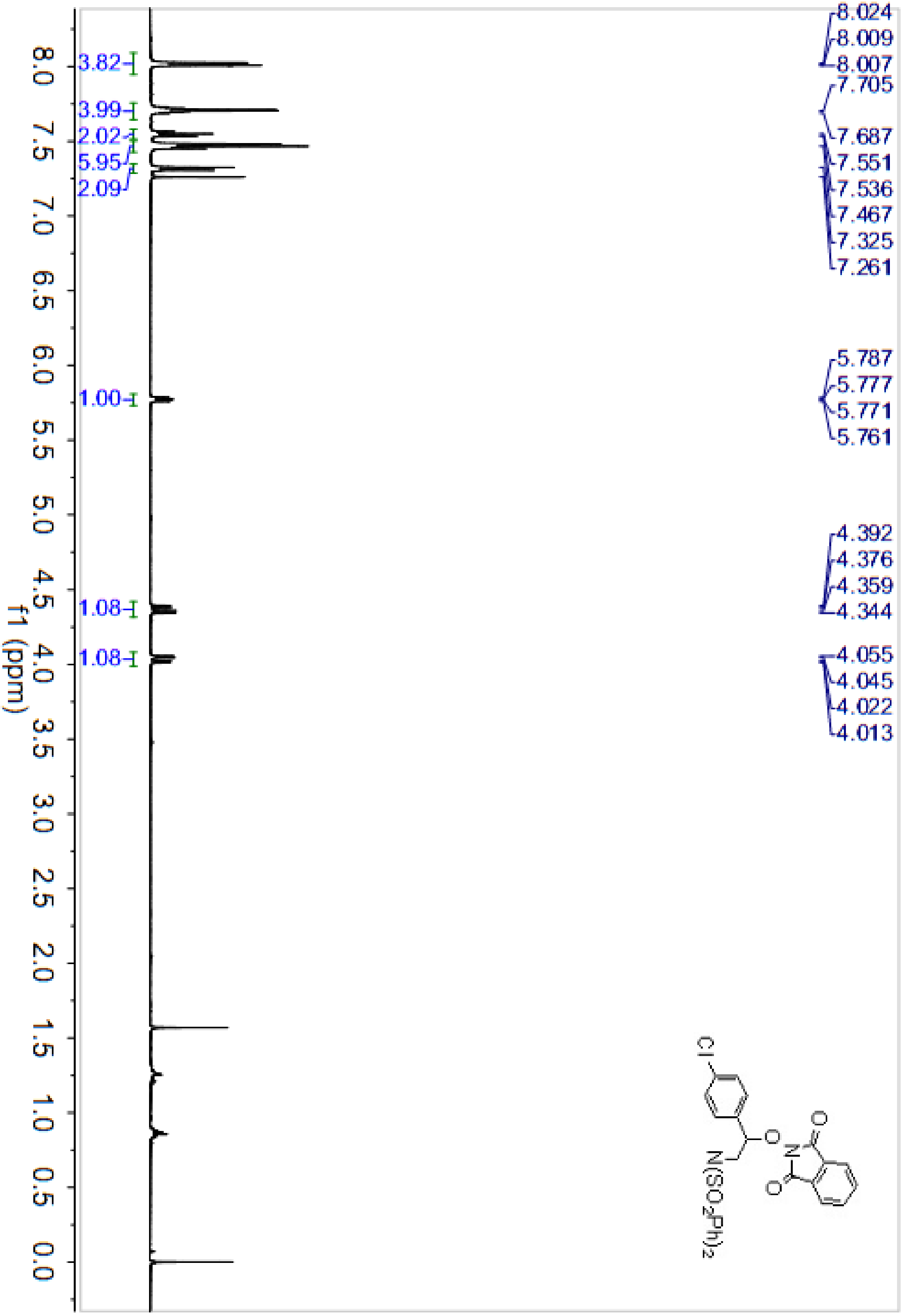

^{13}C NMR of **3a**

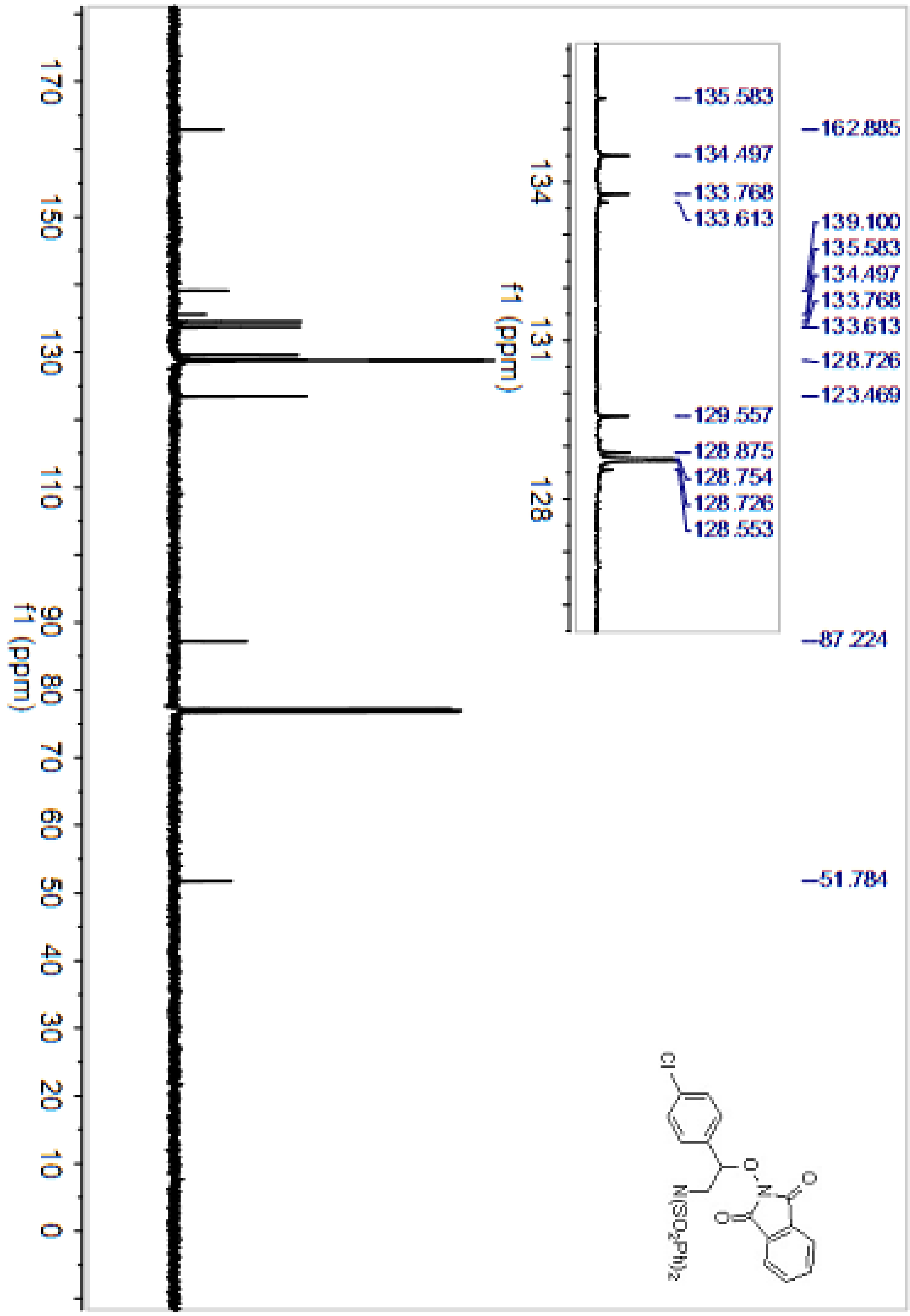
135.583
134.497
133.768
133.613
129.557
128.875
128.754
128.726
128.553
162.885
139.100
135.583
134.497
133.768
133.613
128.726
123.469
87.224
51.784
f1 (ppm)
f1 (ppm)
Cl
O
N
O
O
N(SO2Ph)2

^{1}H MNR of **3b**

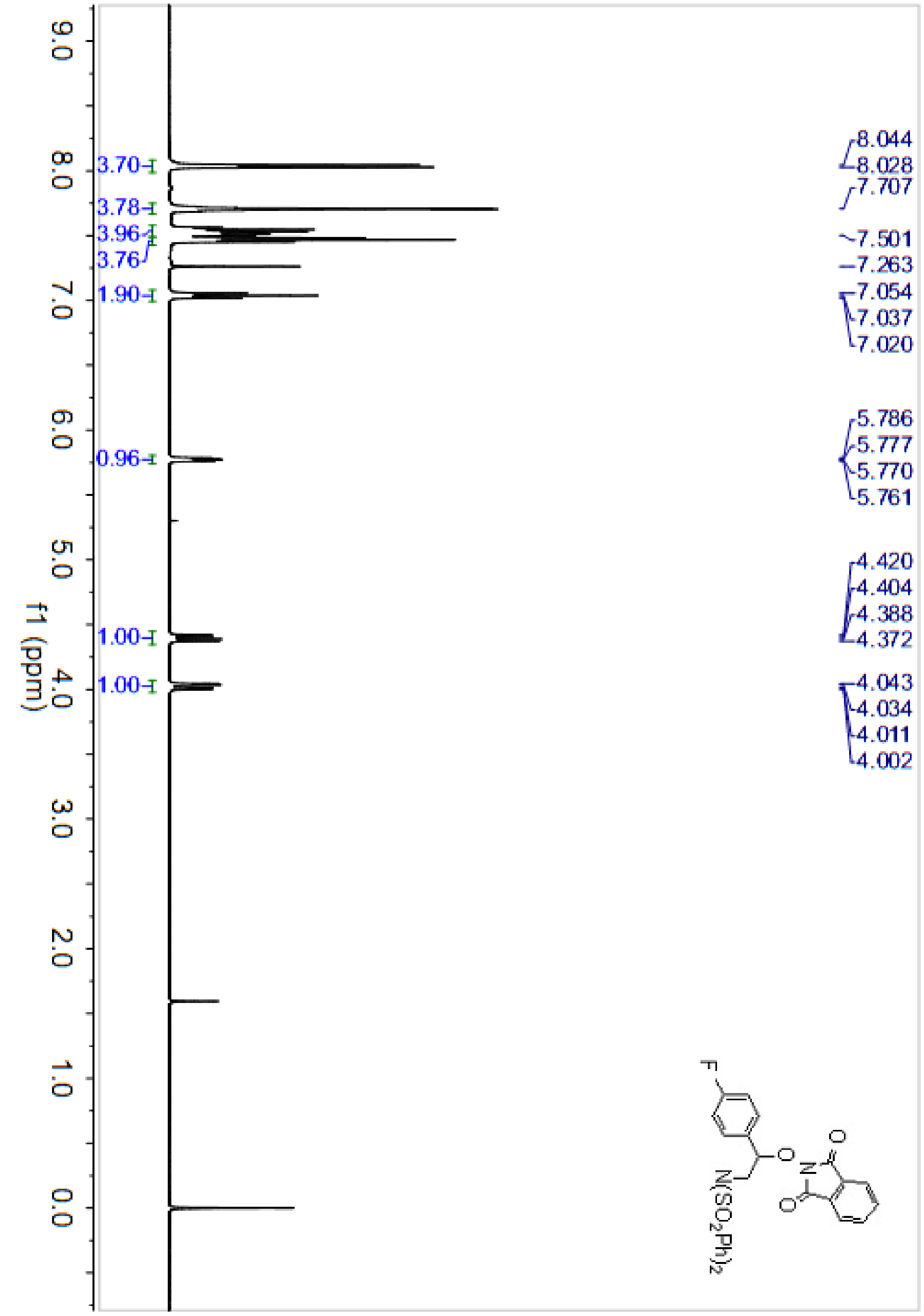

8.044
8.028
7.707
7.501
7.263
7.054
7.037
7.020
5.786
5.777
5.770
5.761
4.420
4.404
4.388
4.372
4.043
4.034
4.011
4.002
3.70
3.78
3.96
3.76
1.90
0.96
1.00
1.00
f1 (ppm)
F
O
N
N(SO2Ph)2

^{13}C NMR of **3b**

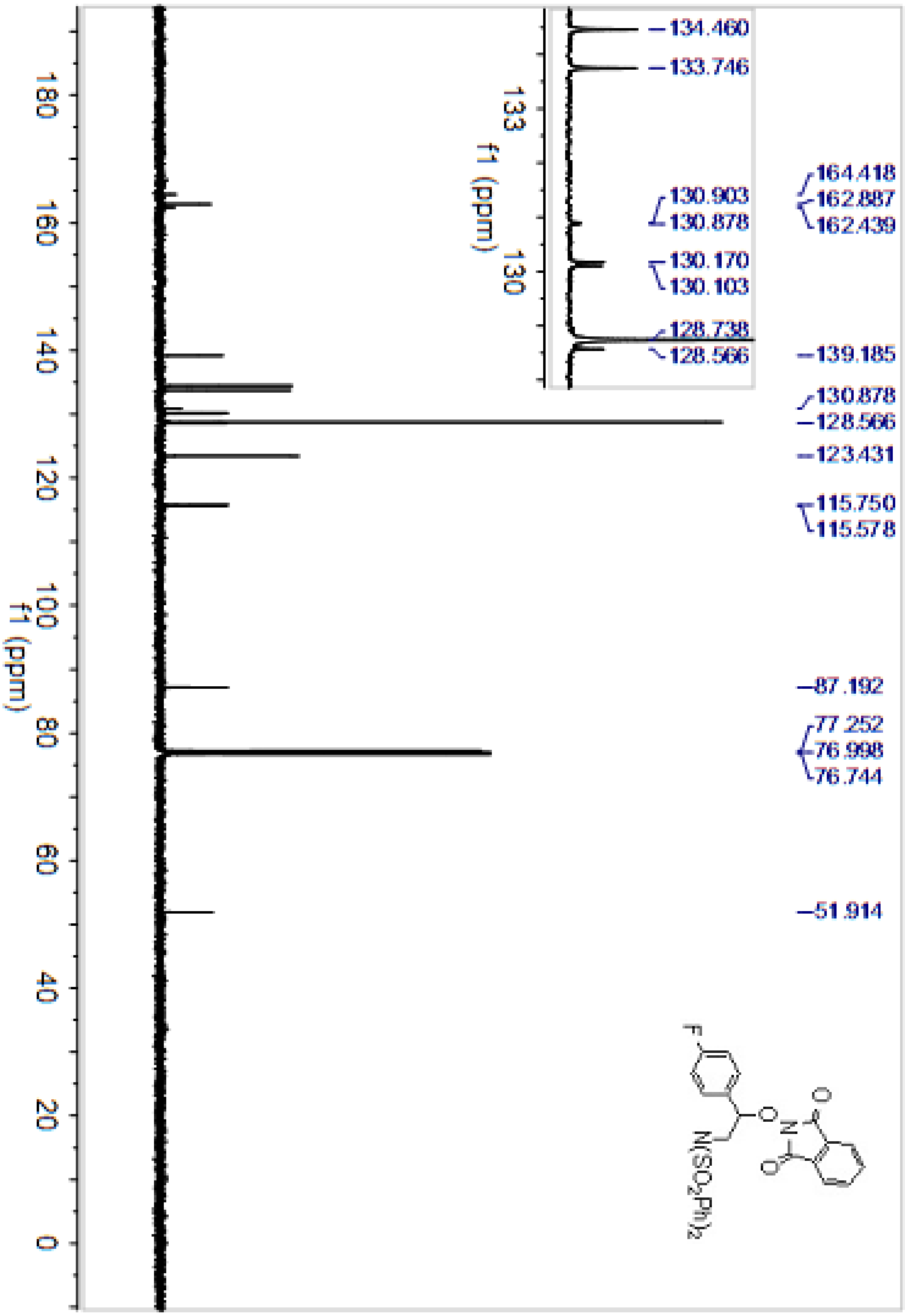
134.460
133.746
130.903
130.878
130.170
130.103
128.738
128.566
164.418
162.887
162.439
139.185
130.878
128.566
123.431
115.750
115.578
87.192
77.252
76.998
76.744
51.914
f1 (ppm)
F
O
N
N(SO2Ph)2

^{1}H NMR of **3c**

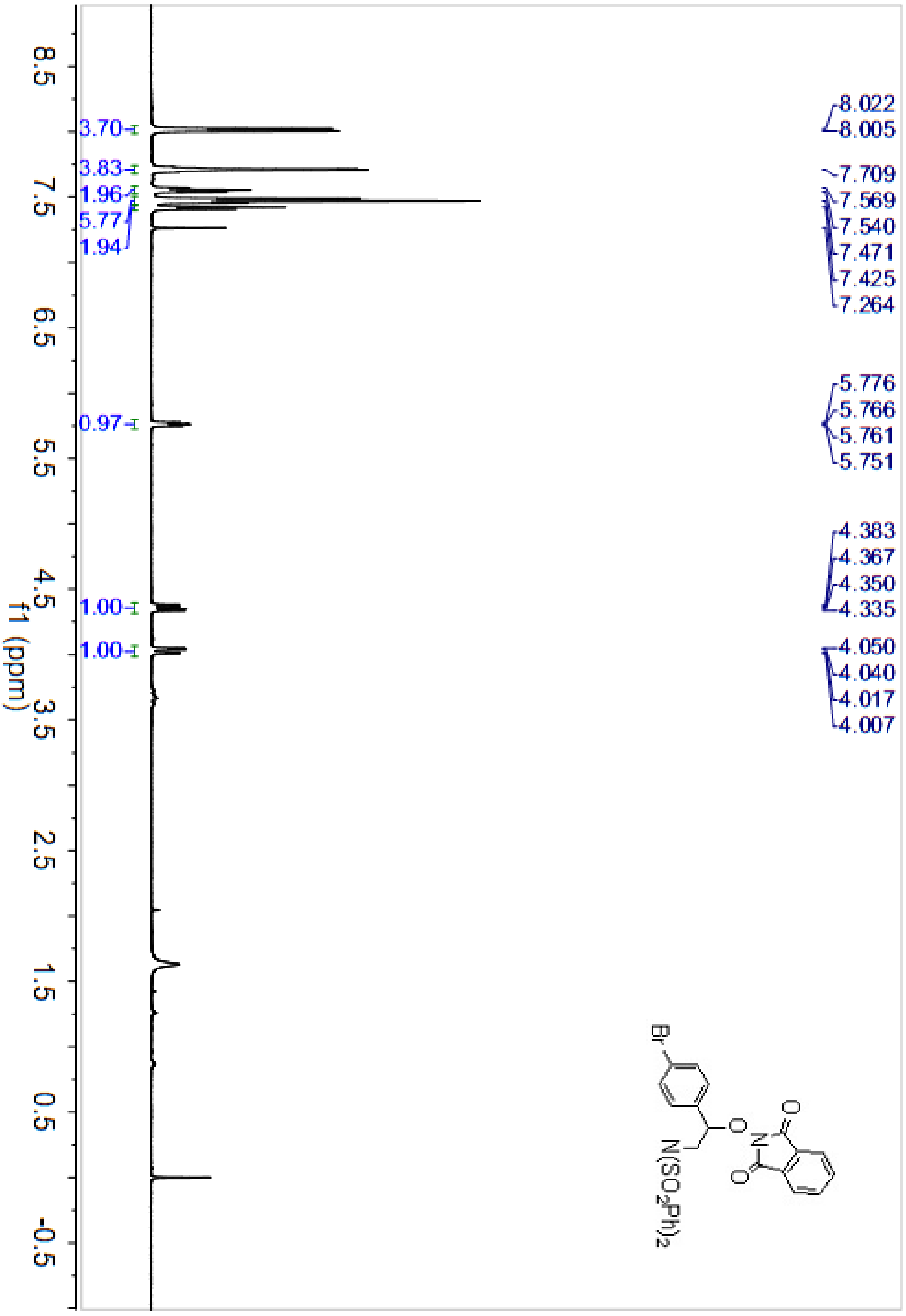
8.022
8.005
7.709
7.569
7.540
7.471
7.425
7.264
5.776
5.766
5.761
5.751
4.383
4.367
4.350
4.335
4.050
4.040
4.017
4.007
3.70
3.83
1.96
5.77
1.94
0.97
1.00
1.00
f1 (ppm)
8.5
7.5
6.5
5.5
4.5
3.5
2.5
1.5
0.5
-0.5
Br
O
N
N(SO2Ph)2

^{13}C NMR of **3c**

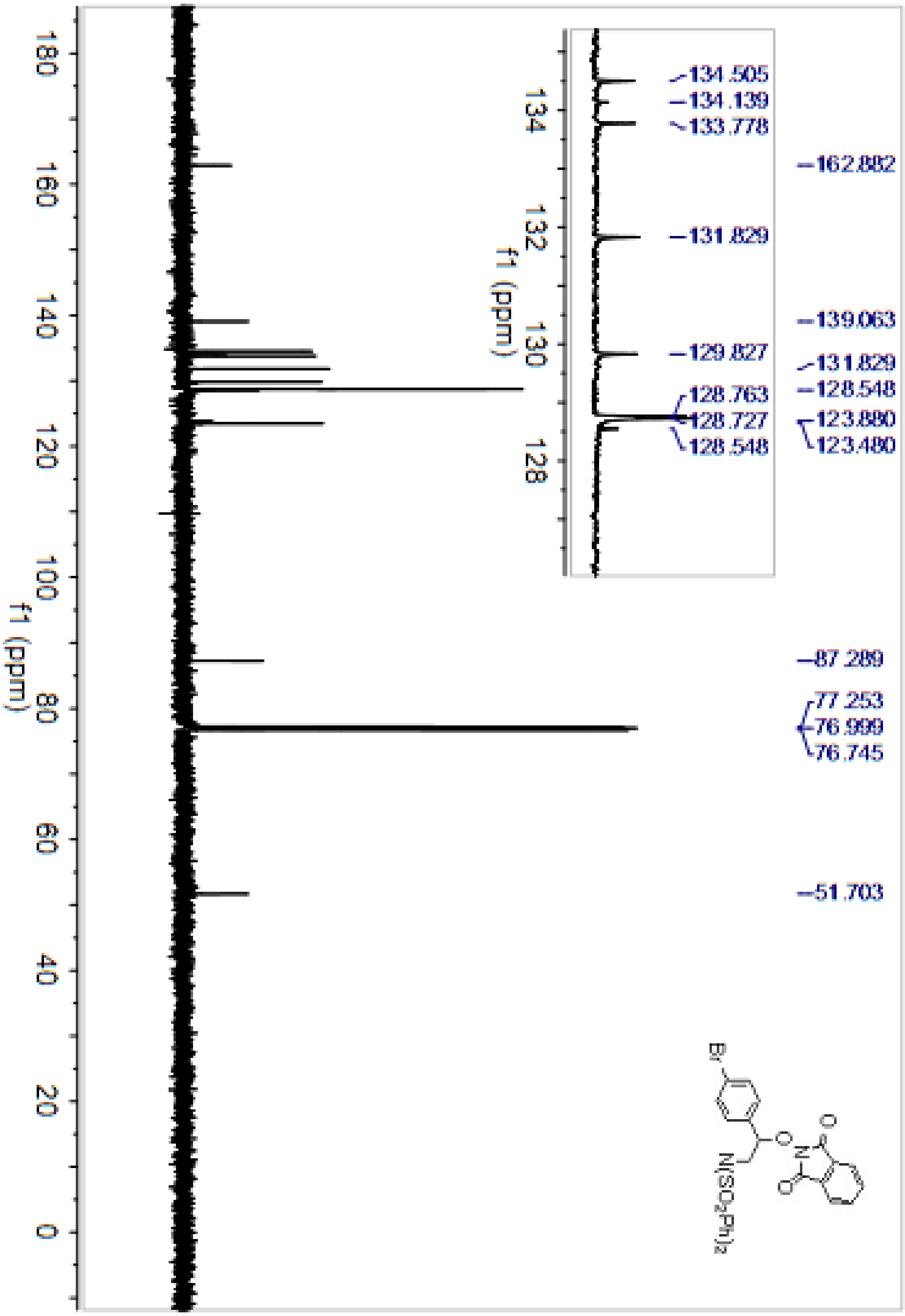
134.505
134.139
133.778
131.829
129.827
128.763
128.727
128.548
f1 (ppm)
162.882
139.063
131.829
128.548
123.880
123.480
87.289
77.253
76.999
76.745
51.703
f1 (ppm)
Br
N(SO$_2$Ph)$_2$

^{1}H NMR of **3d**

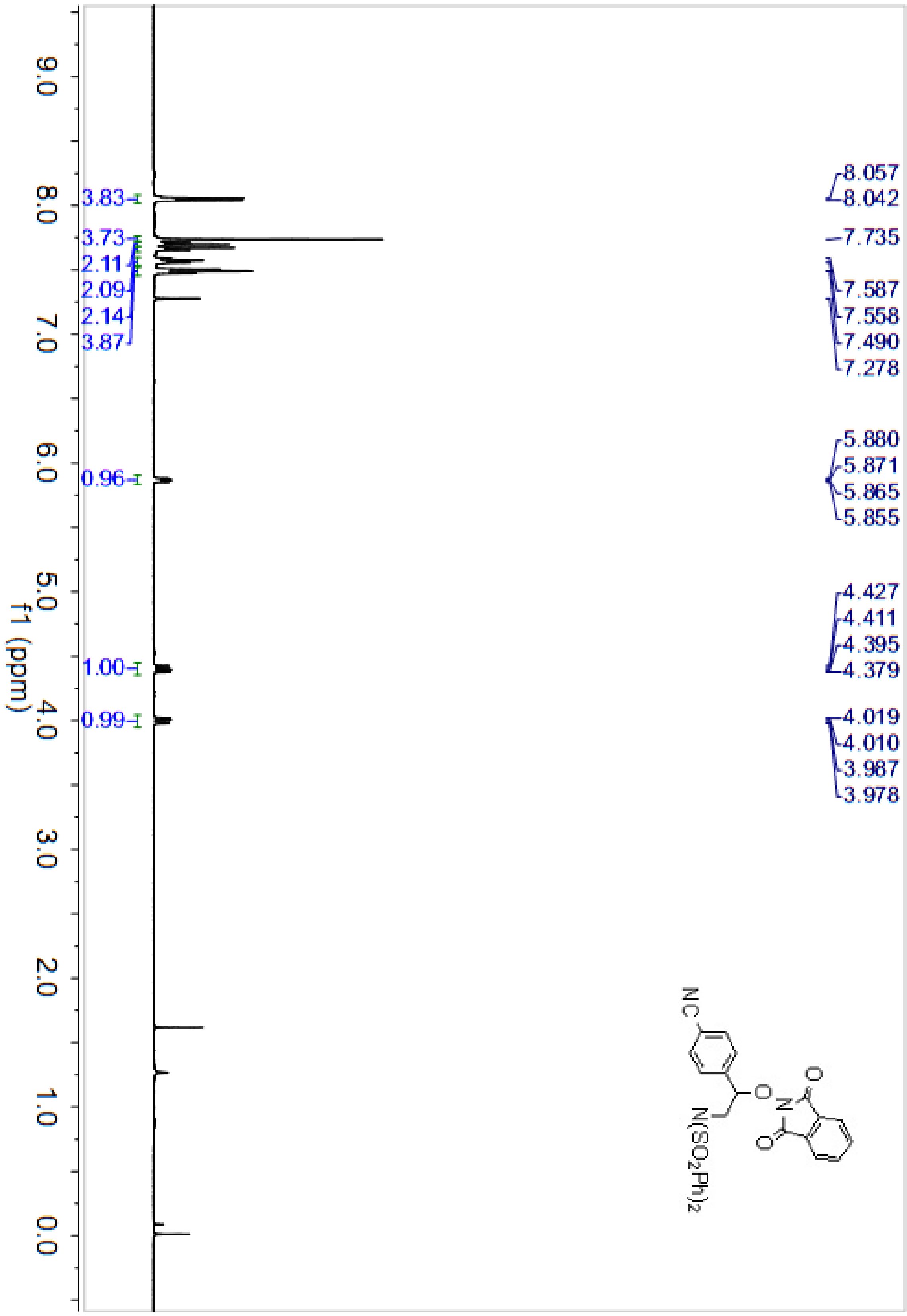

8.057
8.042
7.735
7.587
7.558
7.490
7.278
5.880
5.871
5.865
5.855
4.427
4.411
4.395
4.379
4.019
4.010
3.987
3.978
3.83
3.73
2.11
2.09
2.14
3.87
0.96
1.00
0.99
f1 (ppm)
NC
O
N
N(SO2Ph)2

^{13}C NMR of **3d**

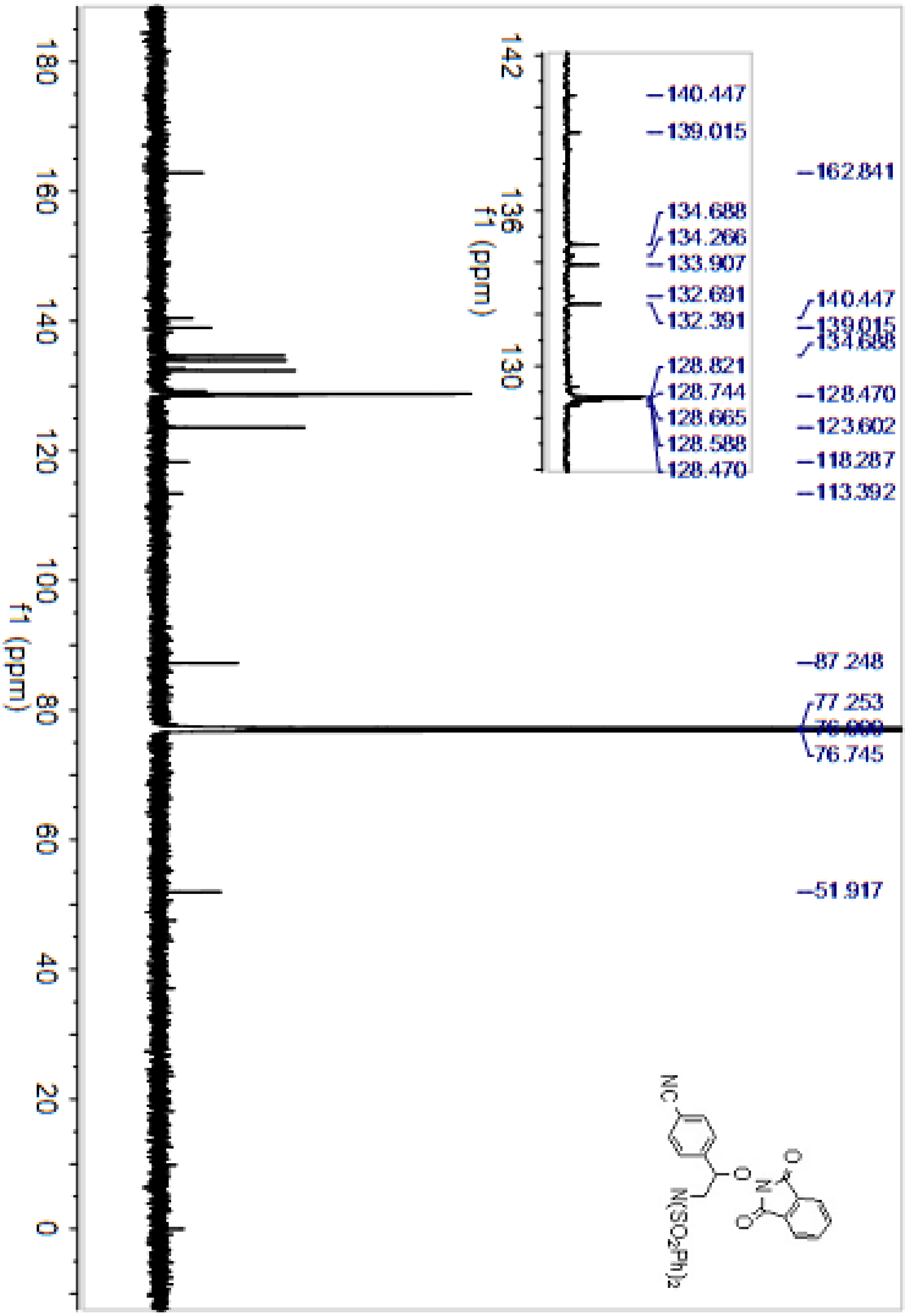

140.447
139.015
134.688
134.266
133.907
132.691
132.391
128.821
128.744
128.665
128.588
128.470
162.841
140.447
139.015
134.688
128.470
123.602
118.287
113.392
87.248
77.253
76.745
51.917
f1 (ppm)
N(SO2Ph)2
NC

[1]H MNR of **3e**

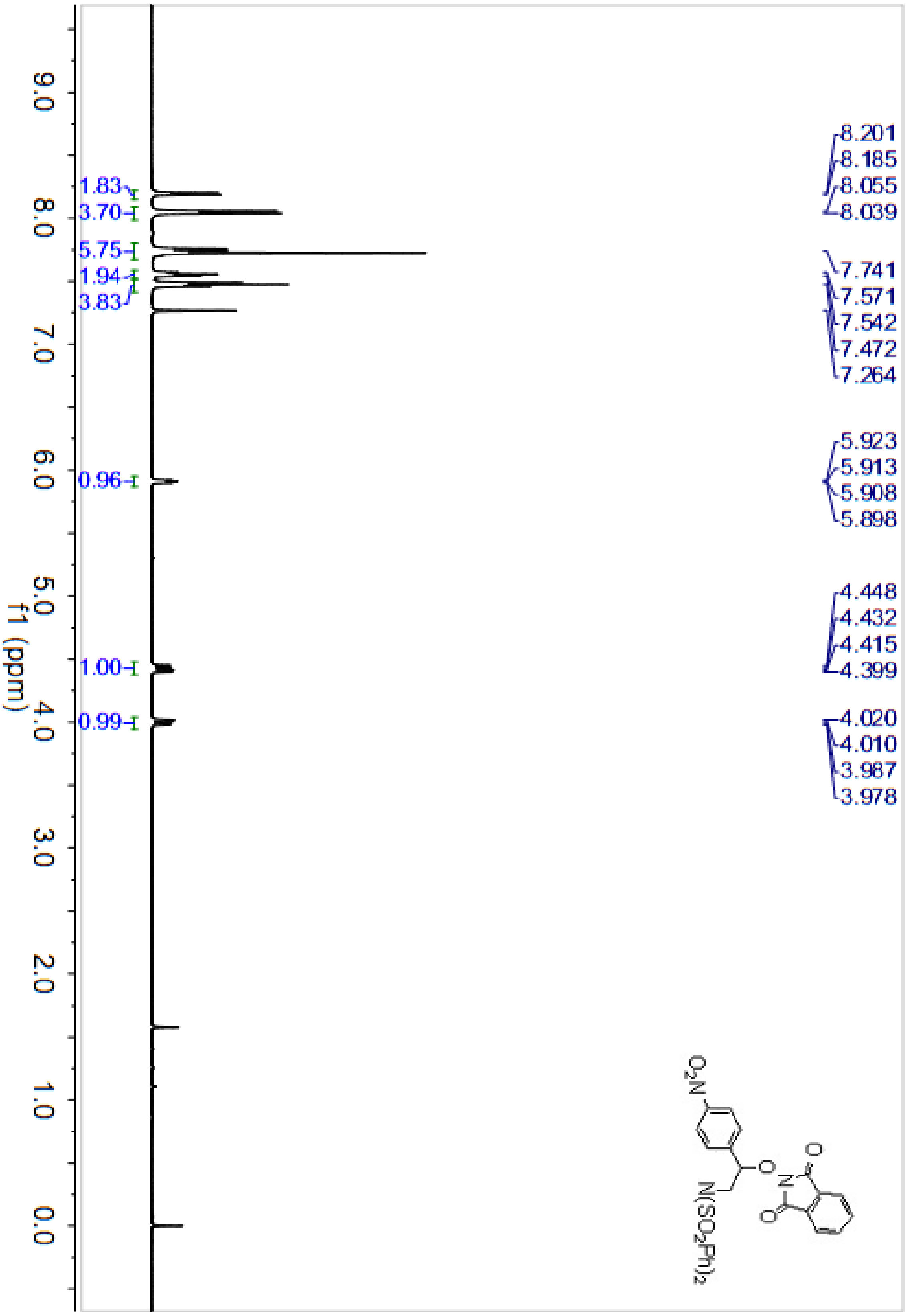
8.201
8.185
8.055
8.039
7.741
7.571
7.542
7.472
7.264
5.923
5.913
5.908
5.898
4.448
4.432
4.415
4.399
4.020
4.010
3.987
3.978
1.83
3.70
5.75
1.94
3.83
0.96
1.00
0.99
f1 (ppm)
O_2N
$N(SO_2Ph)_2$

[13]C MNR of **3e**

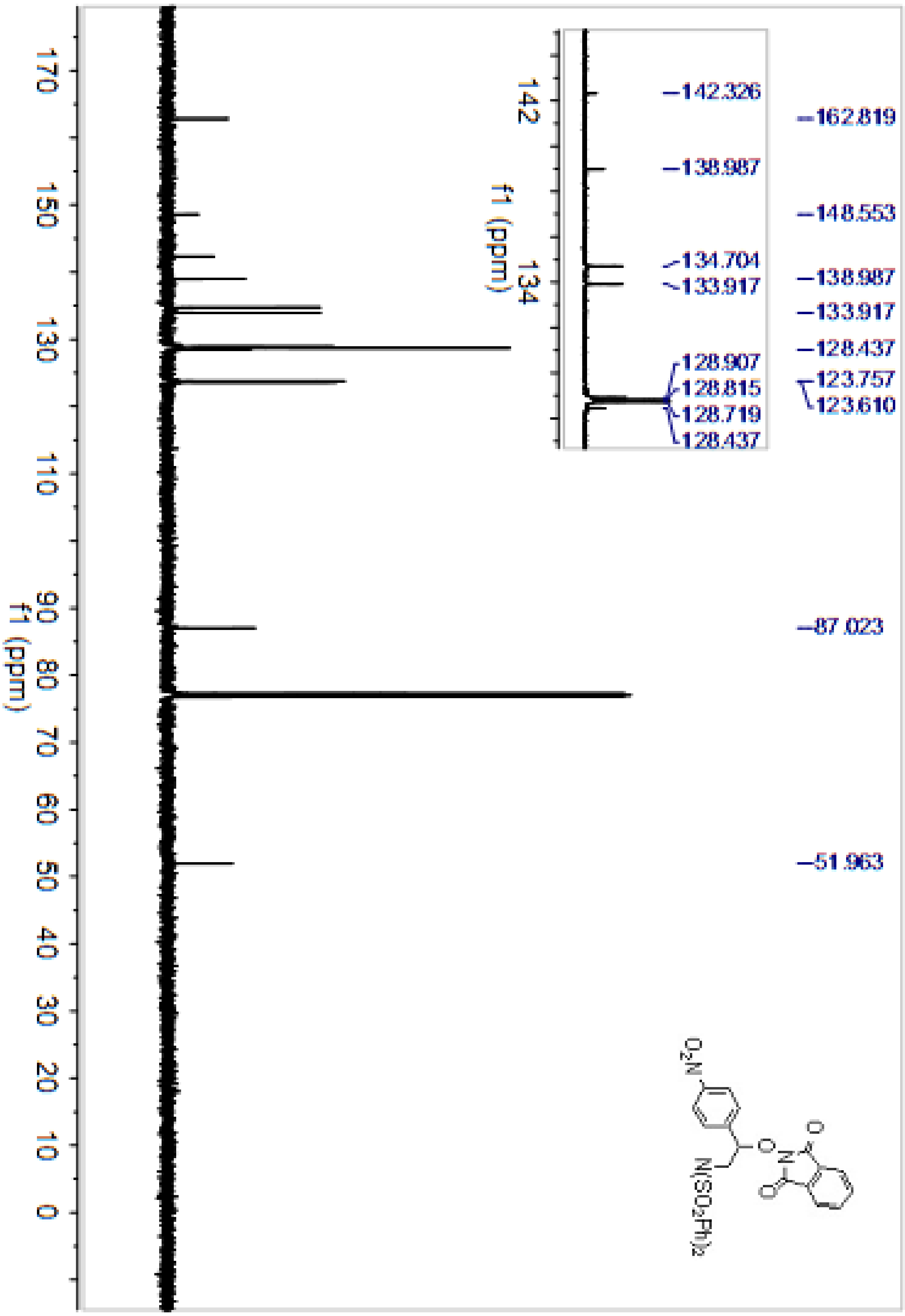
162.819
148.553
138.987
133.917
128.437
123.757
123.610
87.023
51.963
142.326
138.987
134.704
133.917
128.907
128.815
128.719
128.437
f1 (ppm)
O_2N
$N(SO_2Ph)_2$

^{1}H NMR of **3f**

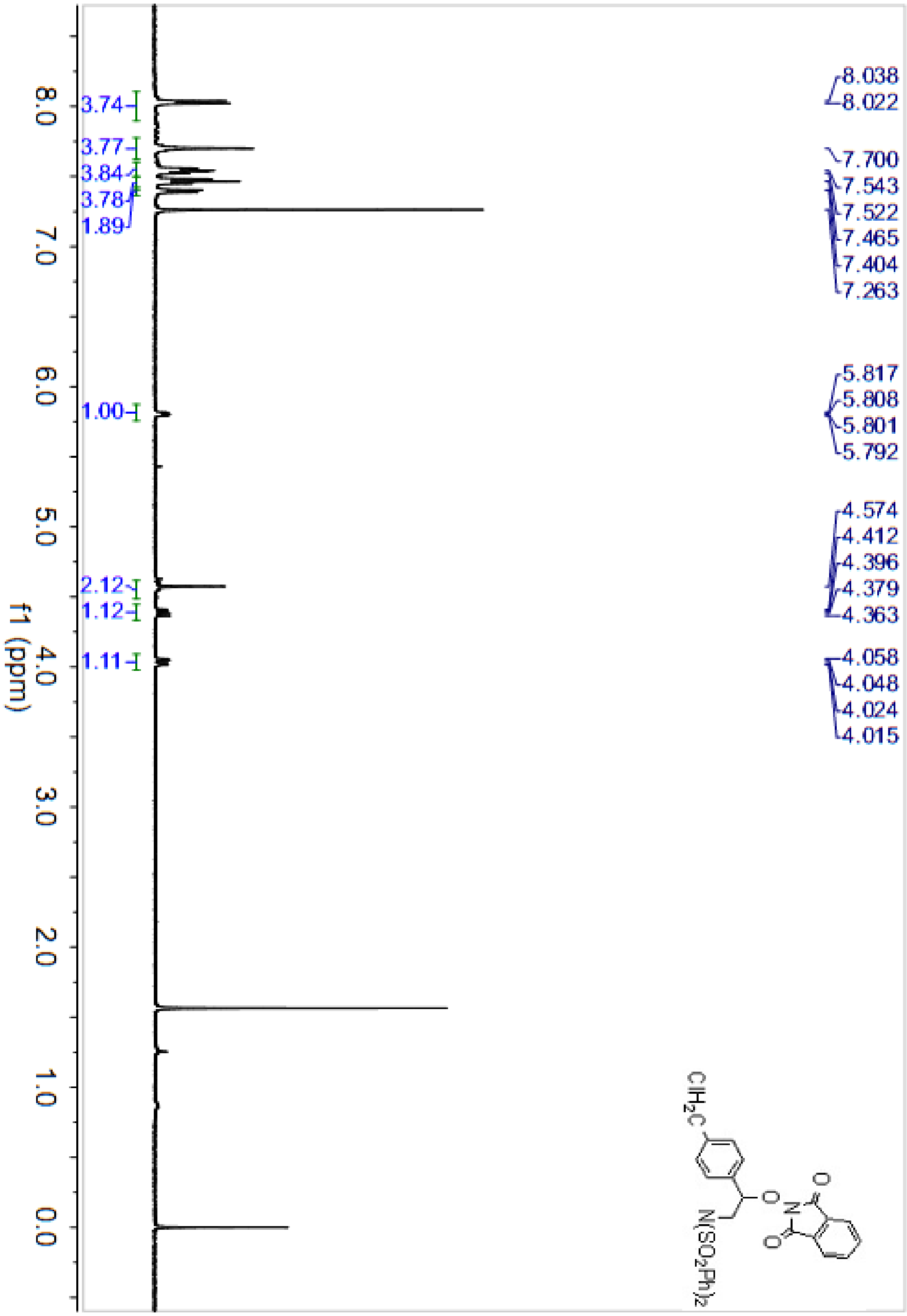

8.038
8.022
7.700
7.543
7.522
7.465
7.404
7.263
5.817
5.808
5.801
5.792
4.574
4.412
4.396
4.379
4.363
4.058
4.048
4.024
4.015
3.74
3.77
3.84
3.78
1.89
1.00
2.12
1.12
1.11
8.0
7.0
6.0
5.0
4.0
3.0
2.0
1.0
0.0
f1 (ppm)
ClH2C
N(SO2Ph)2

^{13}C NMR of **3f**

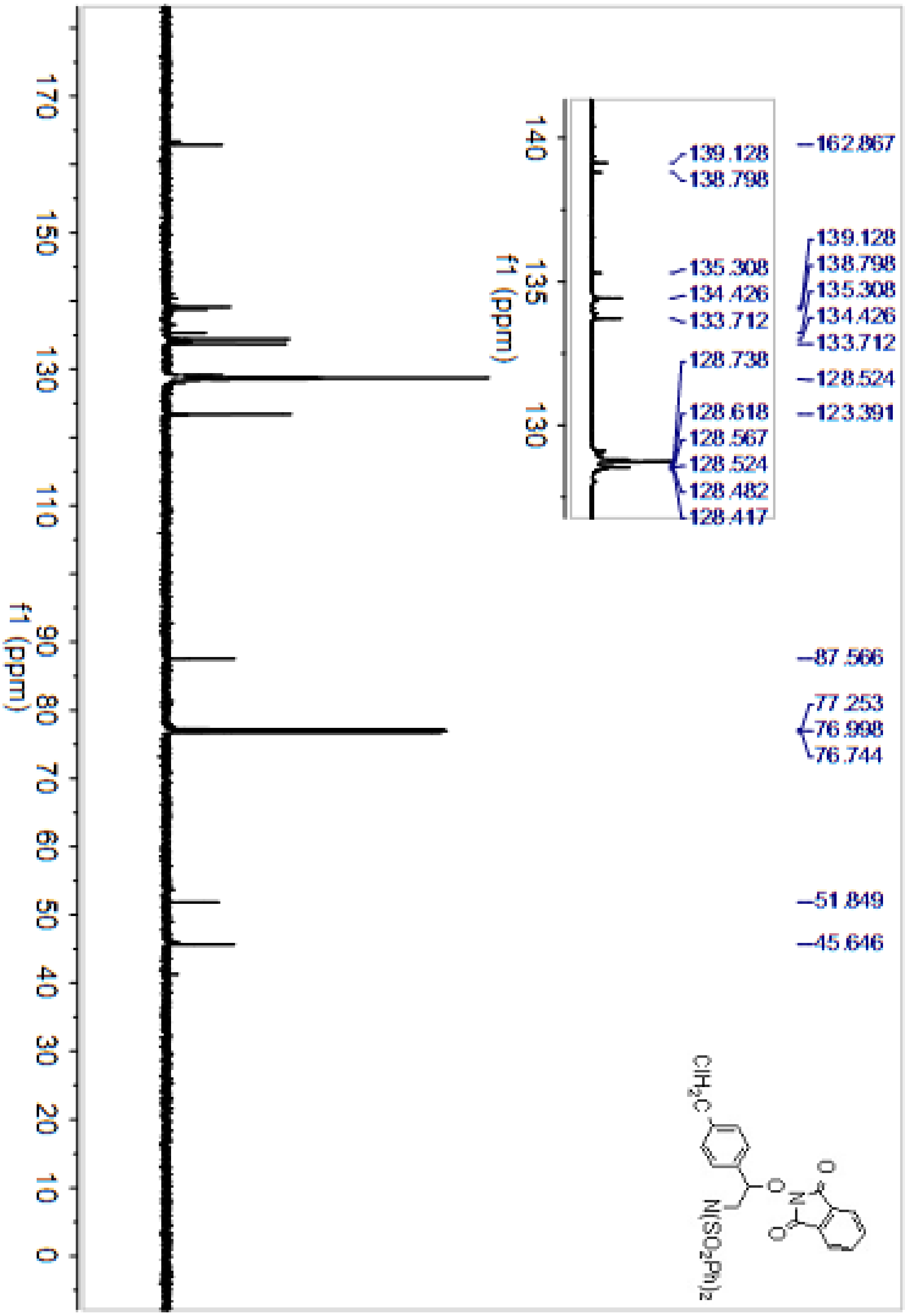
162.867
139.128
138.798
135.308
134.426
133.712
128.524
123.391
87.566
77.253
76.998
76.744
51.849
45.646
128.738
128.618
128.567
128.482
128.417
f1 (ppm)
ClH_2C
$N(SO_2Ph)_2$

^1H NMR of **3g**

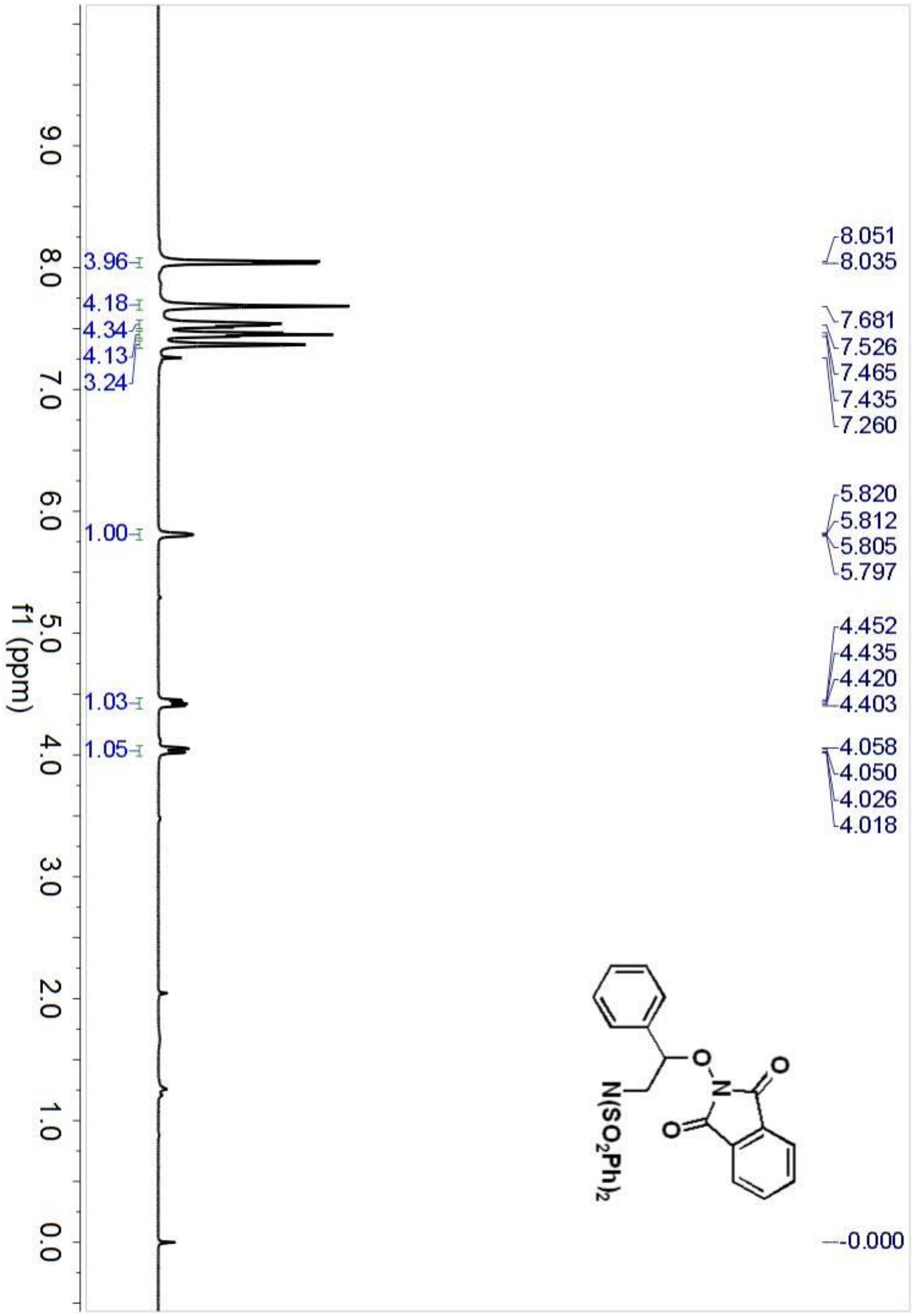

8.051
8.035
7.681
7.526
7.465
7.435
7.260
5.820
5.812
5.805
5.797
4.452
4.435
4.420
4.403
4.058
4.050
4.026
4.018
-0.000
3.96
4.18
4.34
4.13
3.24
1.00
1.03
1.05
f1 (ppm)
9.0
8.0
7.0
6.0
5.0
4.0
3.0
2.0
1.0
0.0
O
N
O
O
N(SO2Ph)2

^{13}C NMR of **3g**

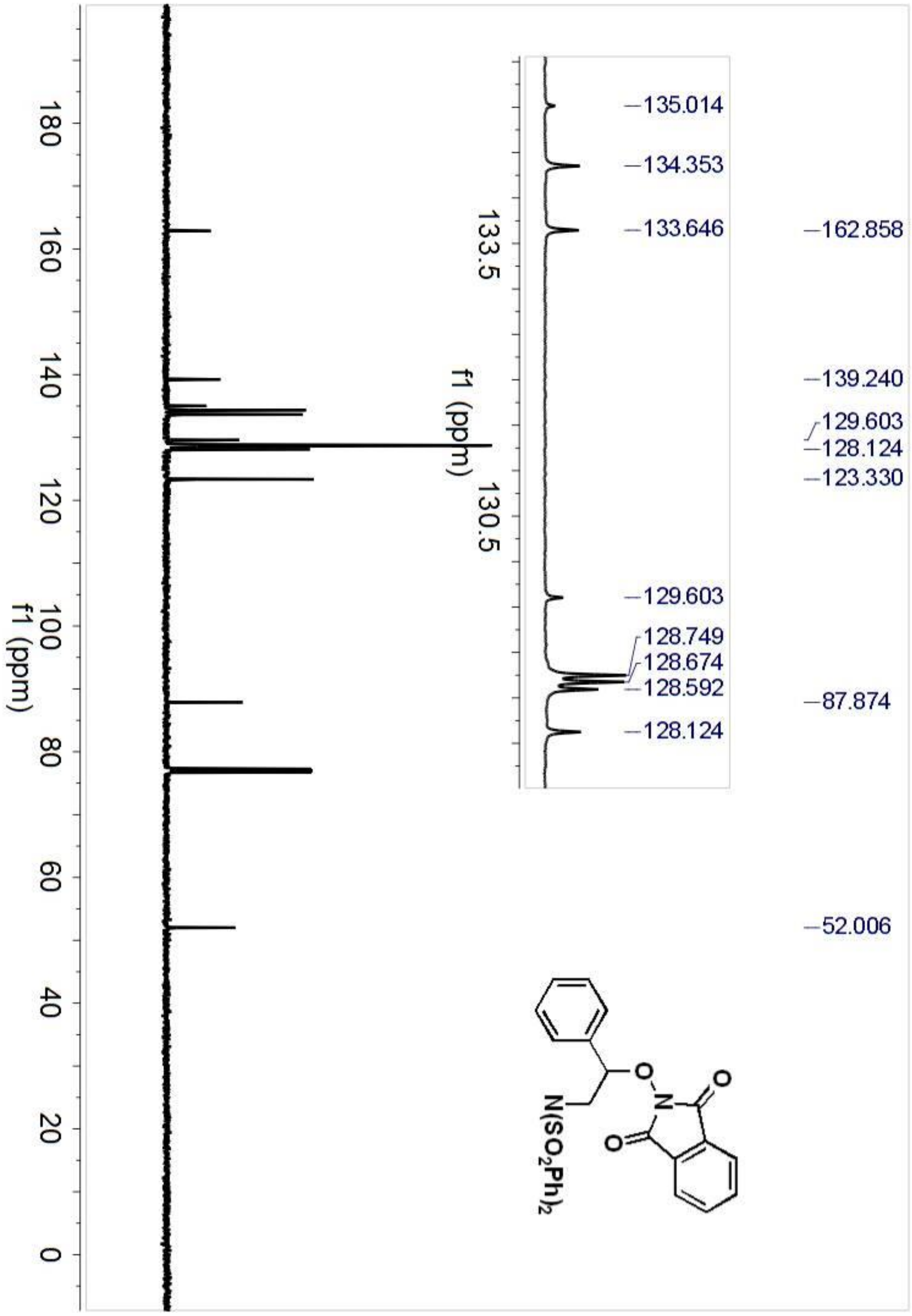

135.014
134.353
133.646
129.603
128.749
128.674
128.592
128.124
133.5
130.5
f1 (ppm)
162.858
139.240
129.603
128.124
123.330
87.874
52.006
180
160
140
120
100
80
60
40
20
0
f1 (ppm)
N
O
N(SO2Ph)2

^{1}H NMR of **3h**

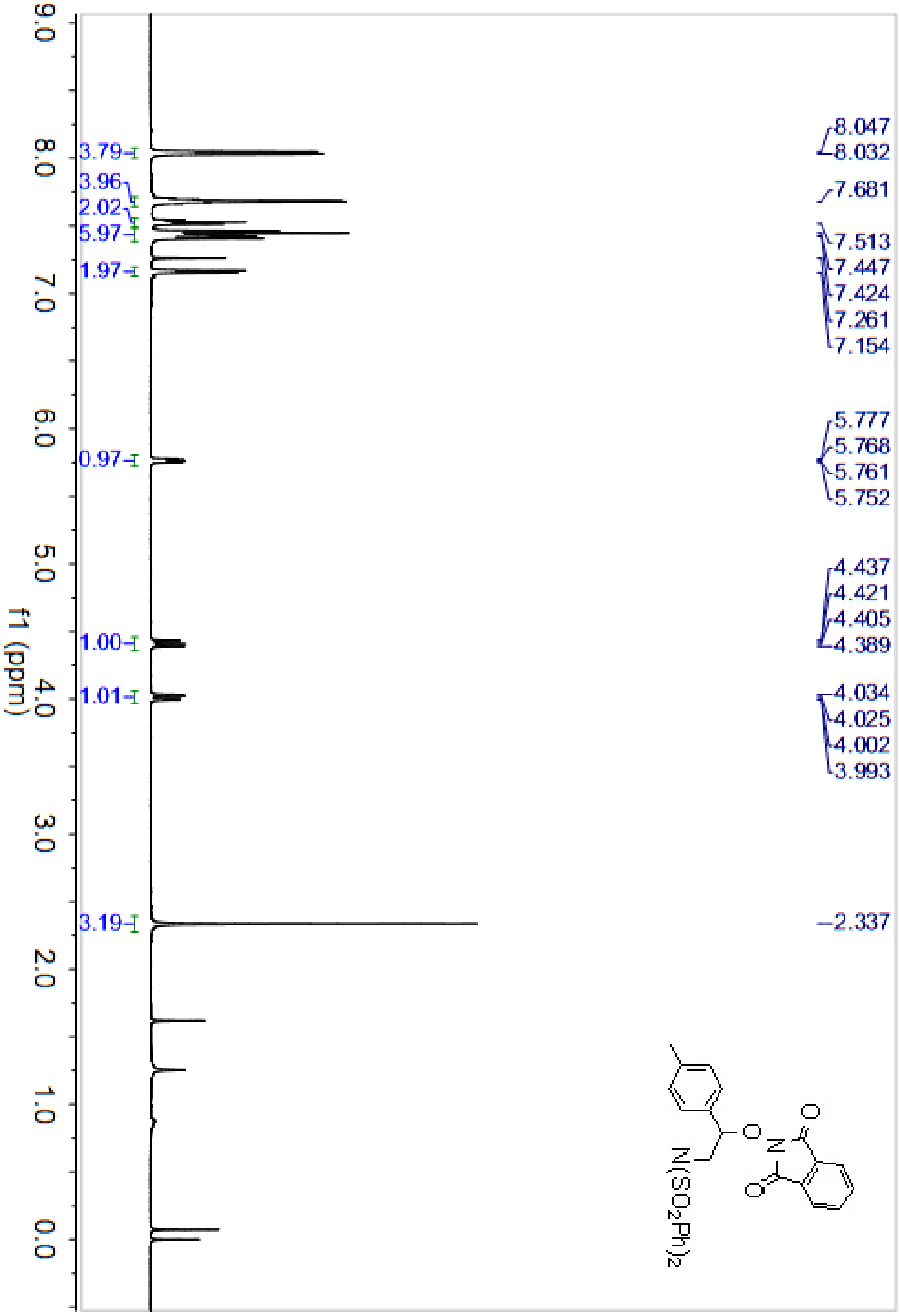

8.047
8.032
7.681
7.513
7.447
7.424
7.261
7.154
5.777
5.768
5.761
5.752
4.437
4.421
4.405
4.389
4.034
4.025
4.002
3.993
2.337
3.79
3.96
2.02
5.97
1.97
0.97
1.00
1.01
3.19
9.0
8.0
7.0
6.0
5.0
4.0
3.0
2.0
1.0
0.0
f1 (ppm)
N(SO2Ph)2

^{13}C NMR of **3h**

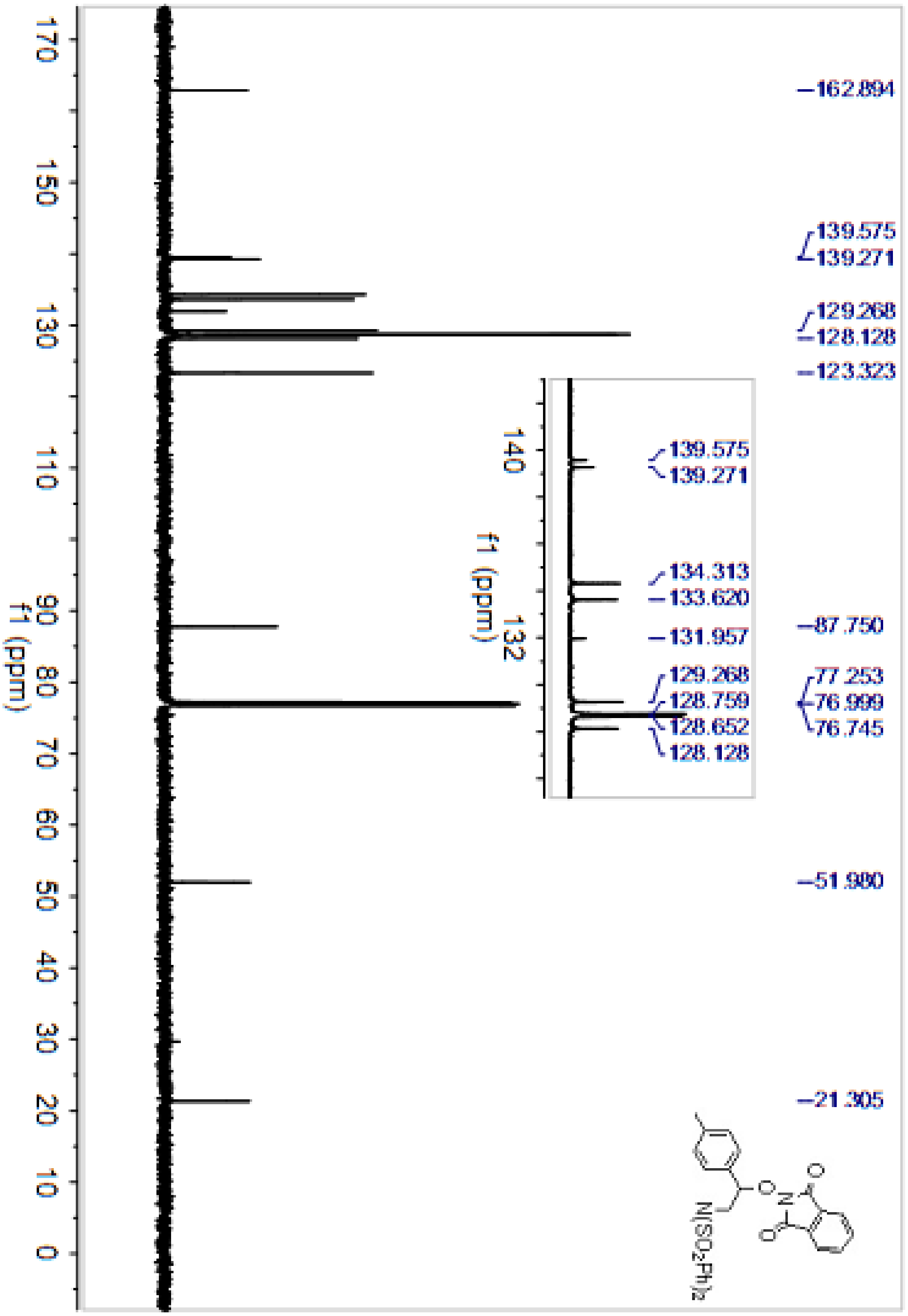
162.894
139.575
139.271
129.268
128.128
123.323
139.575
139.271
134.313
133.620
131.957
129.268
128.759
128.652
128.128
87.750
77.253
76.999
76.745
51.980
21.305
f1 (ppm)
N(SO2Ph)2

^{1}H NMR of **3i**

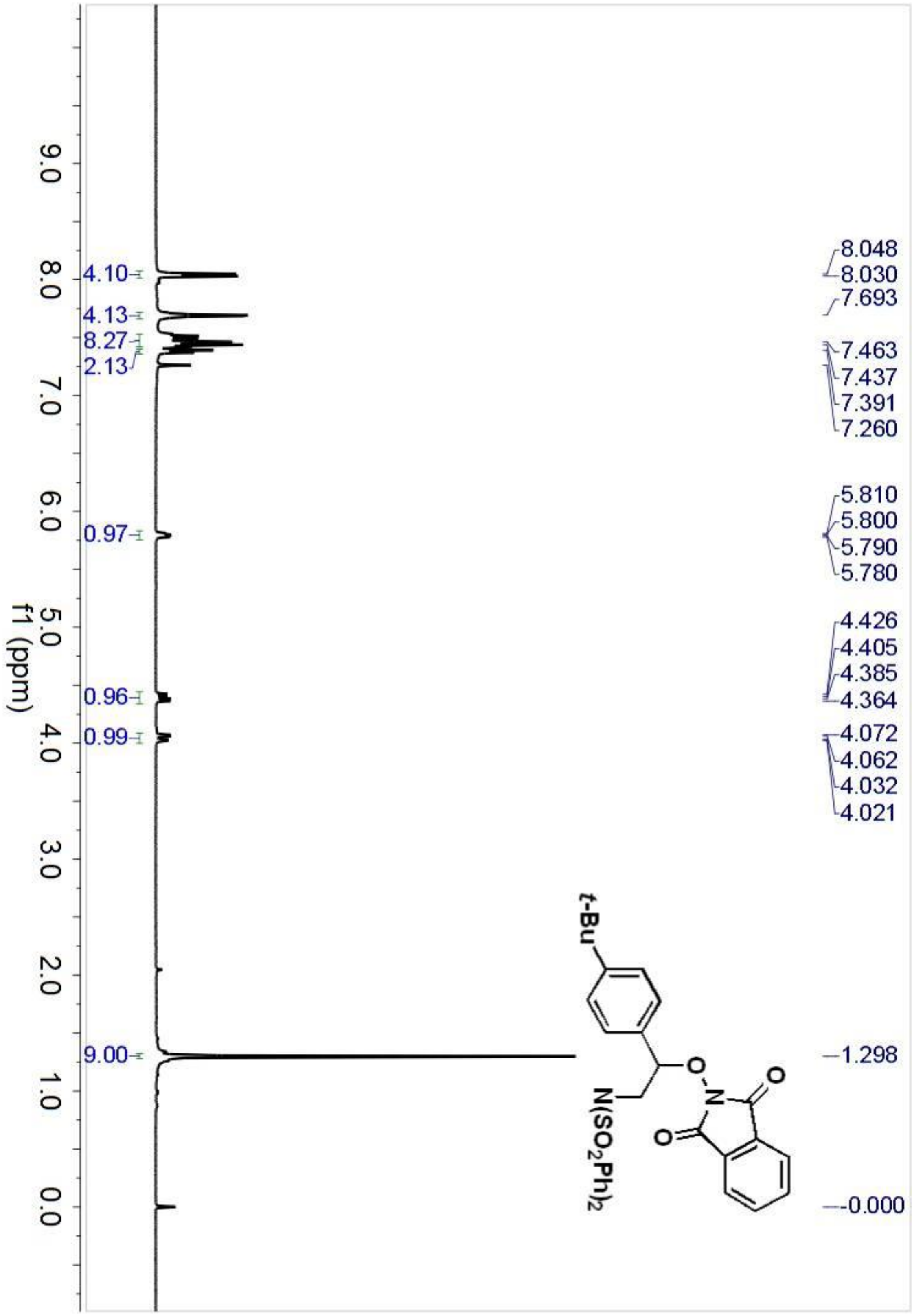
9.0
8.0
7.0
6.0
5.0
4.0
3.0
2.0
1.0
0.0
f1 (ppm)
4.10
4.13
8.27
2.13
0.97
0.96
0.99
9.00
8.048
8.030
7.693
7.463
7.437
7.391
7.260
5.810
5.800
5.790
5.780
4.426
4.405
4.385
4.364
4.072
4.062
4.032
4.021
1.298
-0.000
t-Bu
O
N
O
O
$N(SO_2Ph)_2$

^{13}C NMR of **3i**

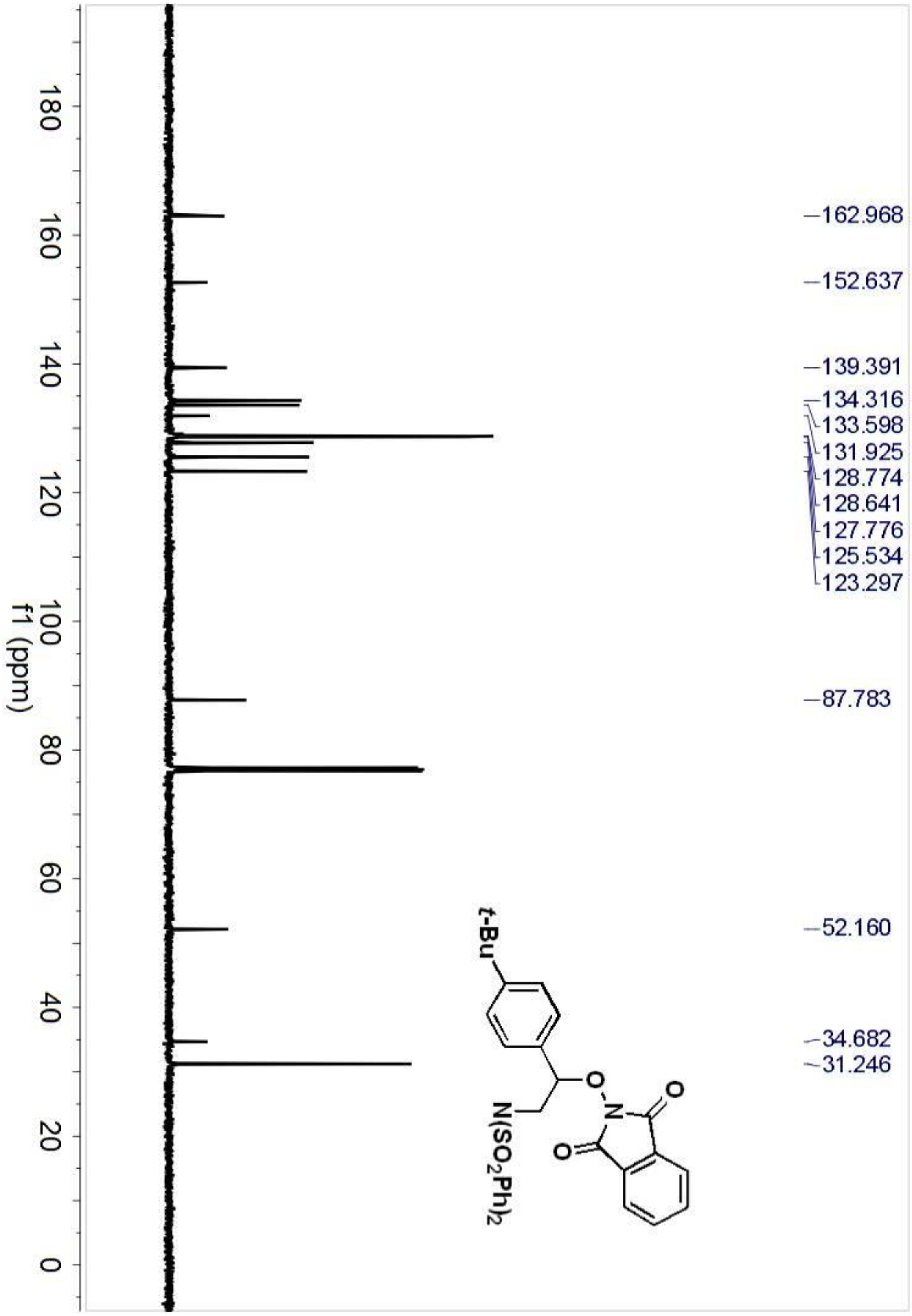
162.968
152.637
139.391
134.316
133.598
131.925
128.774
128.641
127.776
125.534
123.297
87.783
52.160
34.682
31.246
180
160
140
120
100
80
60
40
20
0
f1 (ppm)
t-Bu
O
O
O
N
N(SO2Ph)2

^{1}H NMR of **3j**

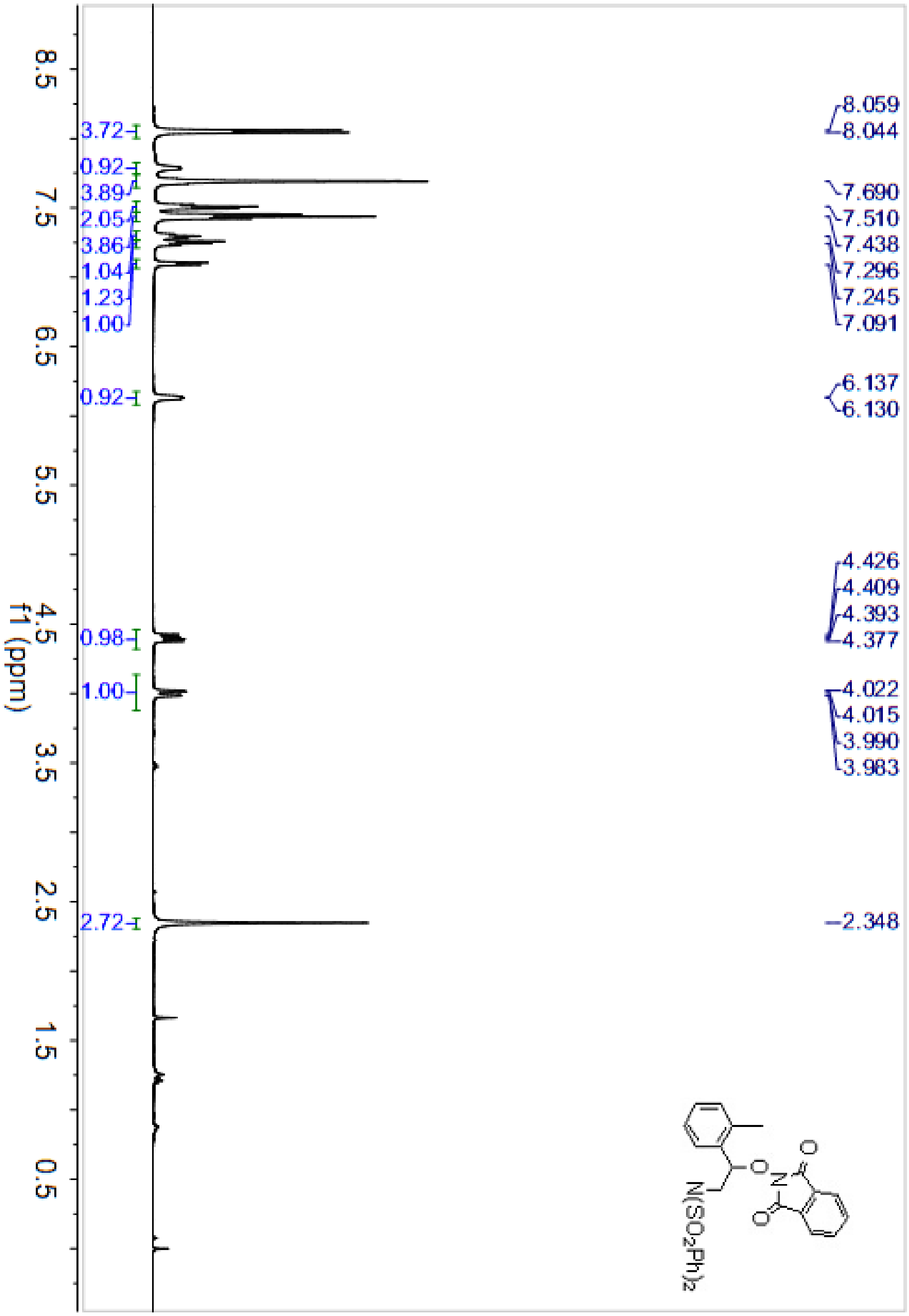

f1 (ppm)
8.5
7.5
6.5
5.5
4.5
3.5
2.5
1.5
0.5
3.72
0.92
3.89
2.05
3.86
1.04
1.23
1.00
0.92
0.98
1.00
2.72
8.059
8.044
7.690
7.510
7.438
7.296
7.245
7.091
6.137
6.130
4.426
4.409
4.393
4.377
4.022
4.015
3.990
3.983
2.348
N(SO2Ph)2

^{13}C NMR of **3j**

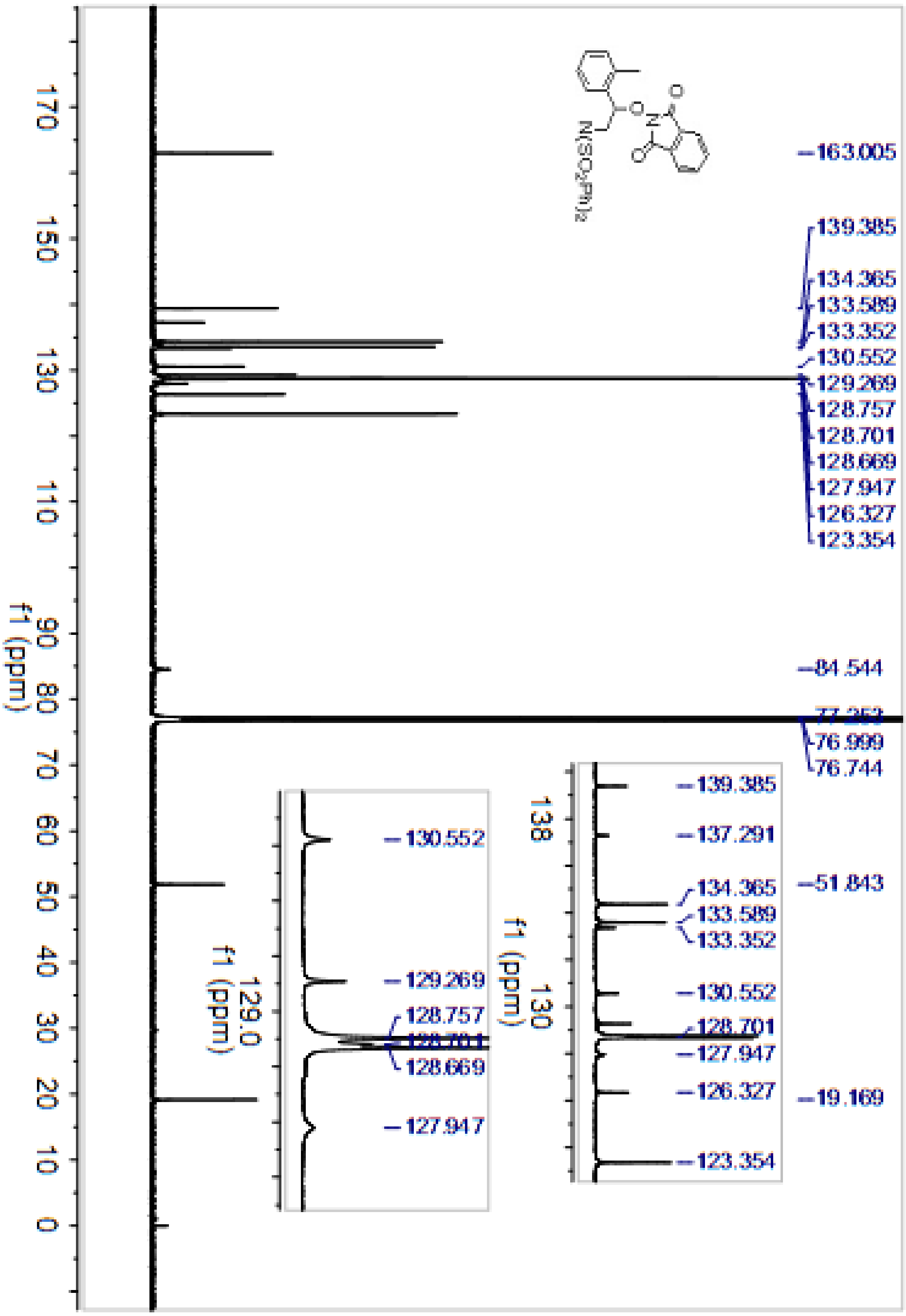

163.005
139.385
134.365
133.589
133.352
130.552
129.269
128.757
128.701
128.669
127.947
126.327
123.354
84.544
76.999
76.744
51.843
19.169
137.291
f1 (ppm)
N(SO2Ph)2

^{1}H NMR of **3k**

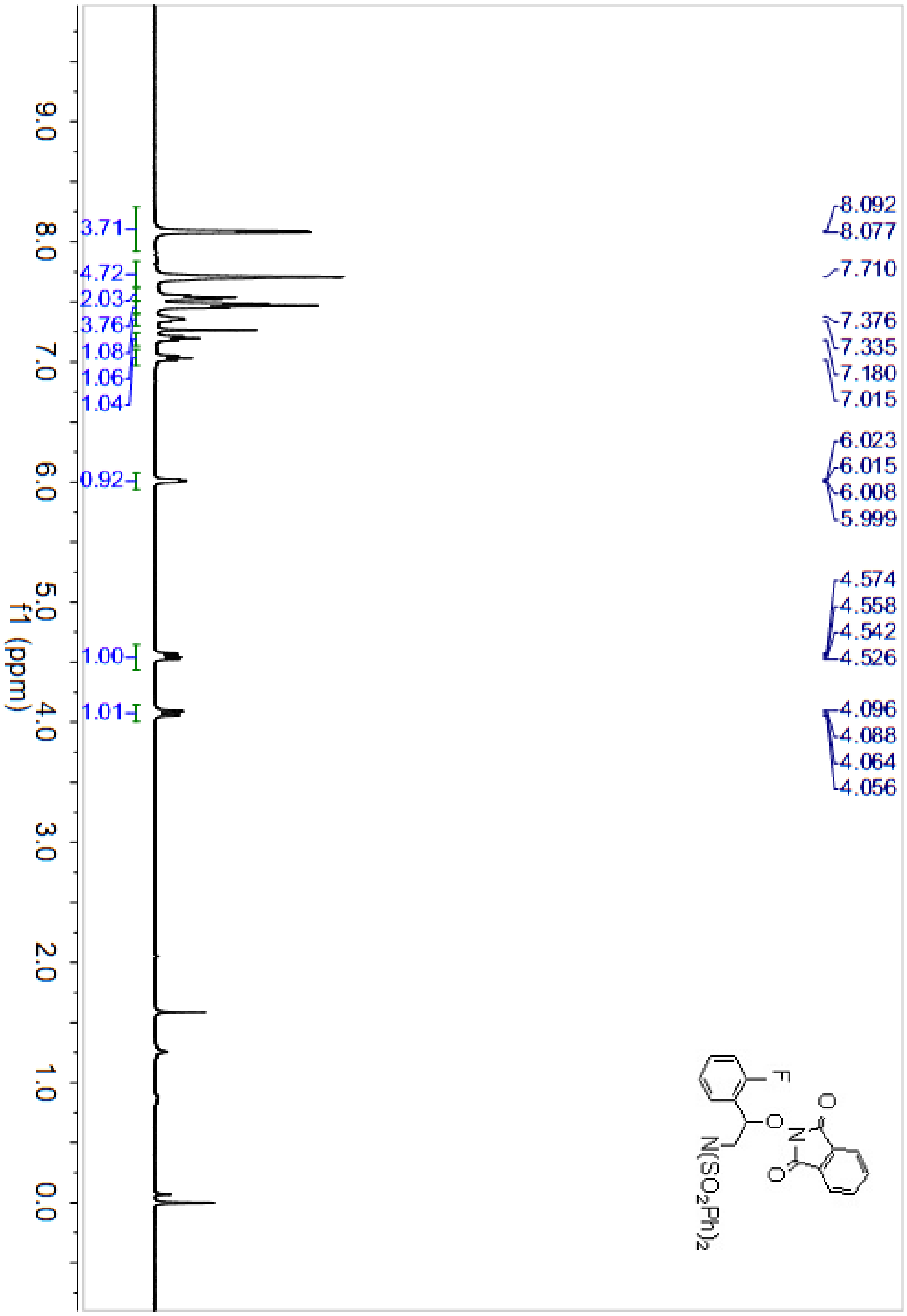
8.092
8.077
7.710
7.376
7.335
7.180
7.015
6.023
6.015
6.008
5.999
4.574
4.558
4.542
4.526
4.096
4.088
4.064
4.056
3.71
4.72
2.03
3.76
1.08
1.06
1.04
0.92
1.00
1.01
9.0
8.0
7.0
6.0
5.0
4.0
3.0
2.0
1.0
0.0
f1 (ppm)
F
O
N
$N(SO_2Ph)_2$

^{13}C NMR of **3k**

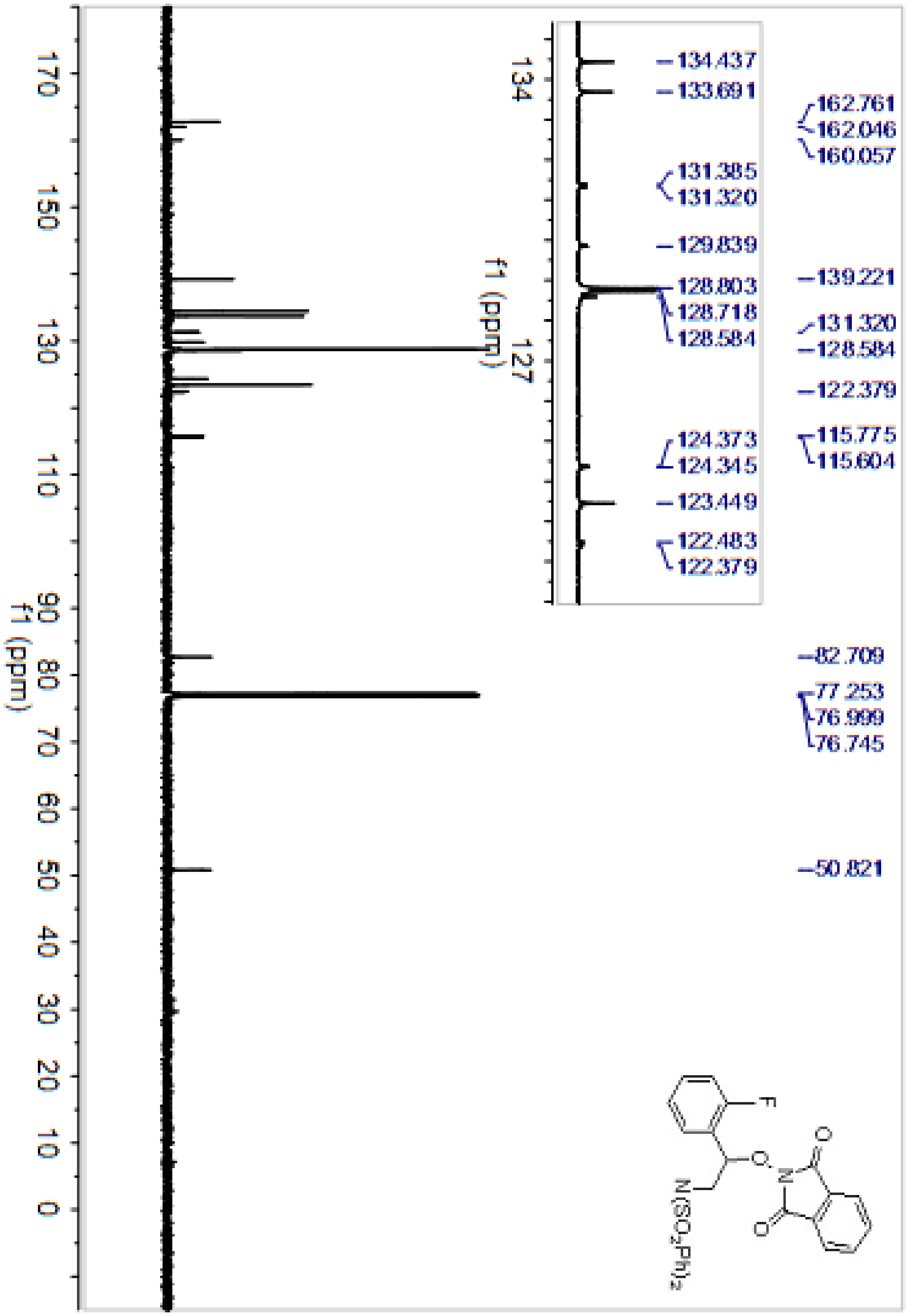
134.437
133.691
131.385
131.320
129.839
128.803
128.718
128.584
124.373
124.345
123.449
122.483
122.379
162.761
162.046
160.057
139.221
131.320
128.584
122.379
115.775
115.604
82.709
77.253
76.999
76.745
50.821
f1 (ppm)
F
O
N
N(SO2Ph)2

^{1}H NMR **3l**

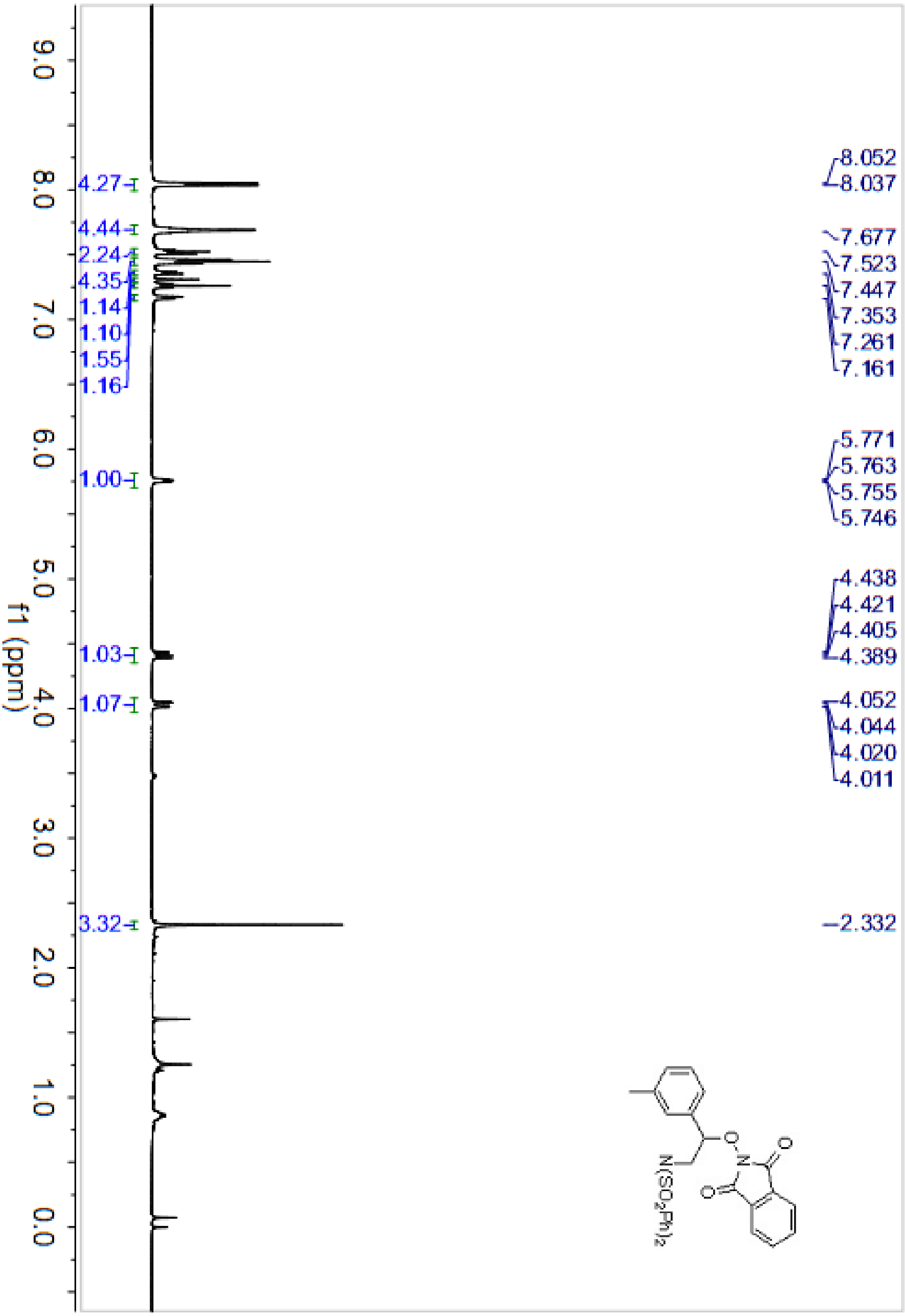

f1 (ppm)
8.052
8.037
7.677
7.523
7.447
7.353
7.261
7.161
5.771
5.763
5.755
5.746
4.438
4.421
4.405
4.389
4.052
4.044
4.020
4.011
2.332
4.27
4.44
2.24
4.35
1.14
1.10
1.55
1.16
1.00
1.03
1.07
3.32
N(SO2Ph)2

^{13}C NMR **3l**

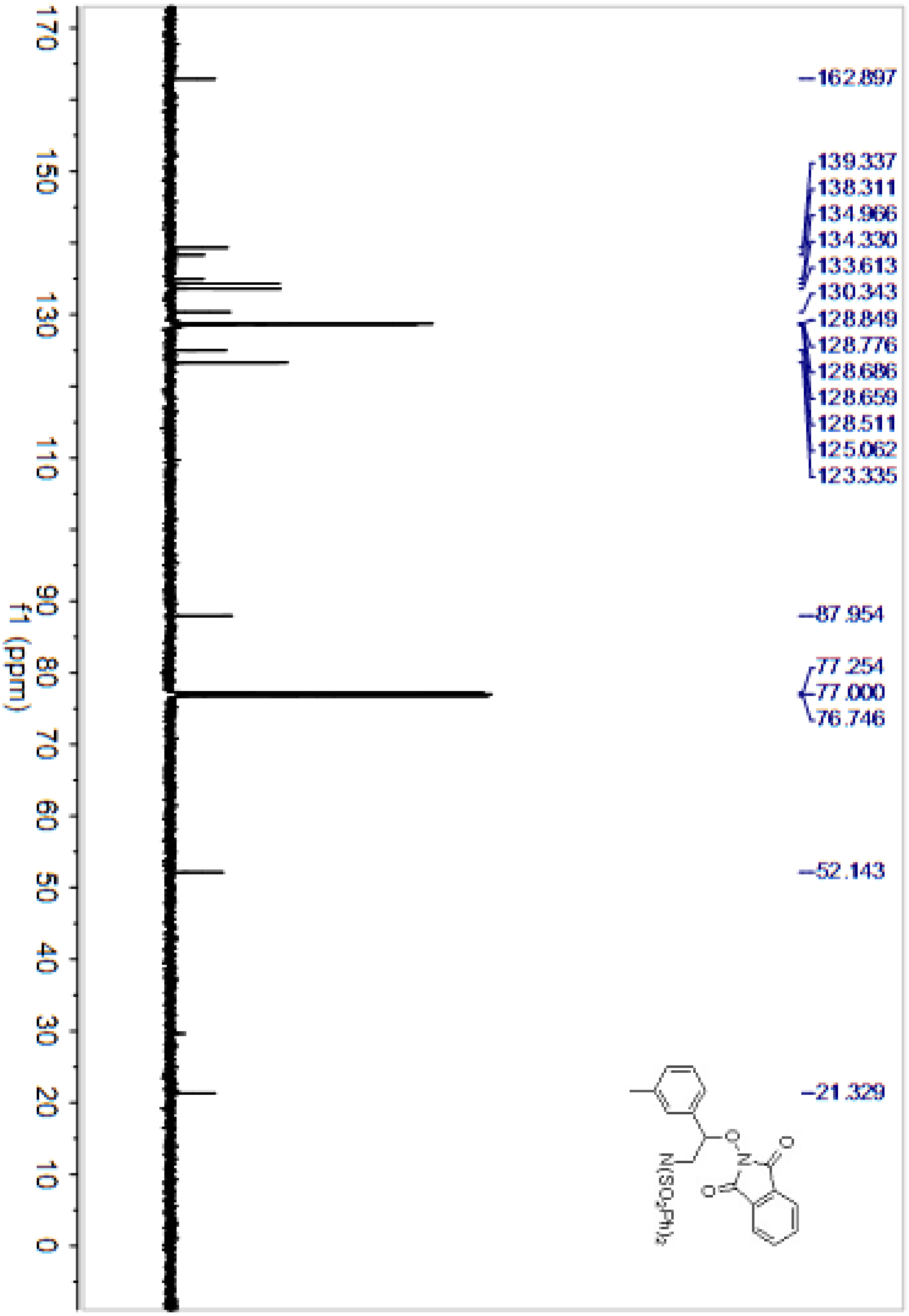
162.897
139.337
138.311
134.966
134.330
133.613
130.343
128.849
128.776
128.686
128.659
128.511
125.062
123.335
87.954
77.254
77.000
76.746
52.143
21.329
f1 (ppm)
N(SO2Ph)2

^{1}H NMR of **3m**

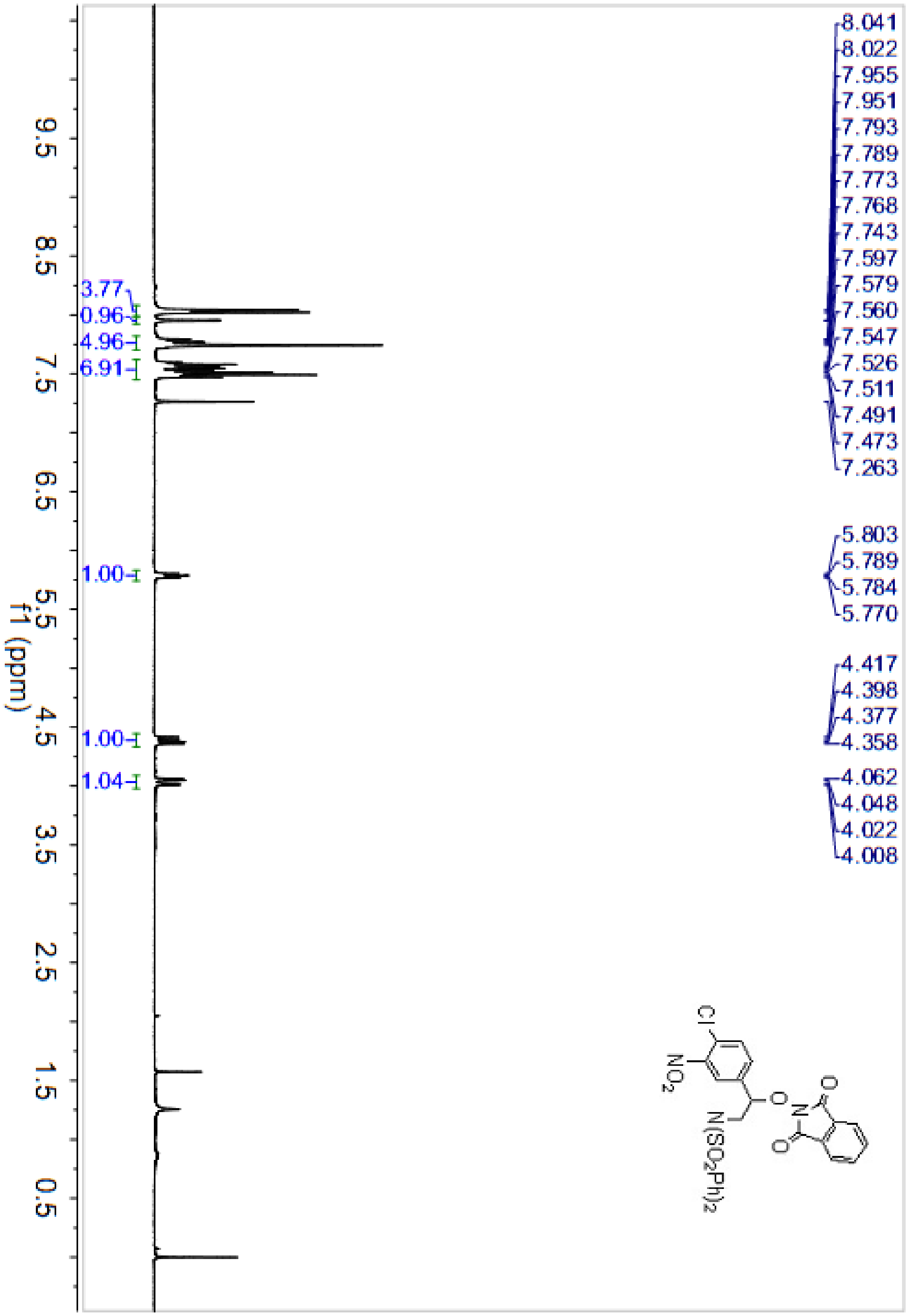
8.041
8.022
7.955
7.951
7.793
7.789
7.773
7.768
7.743
7.597
7.579
7.560
7.547
7.526
7.511
7.491
7.473
7.263
5.803
5.789
5.784
5.770
4.417
4.398
4.377
4.358
4.062
4.048
4.022
4.008
3.77
0.96
4.96
6.91
1.00
1.00
1.04
9.5
8.5
7.5
6.5
5.5
4.5
3.5
2.5
1.5
0.5
f1 (ppm)
N(SO2Ph)2
NO2
Cl

^{13}C NMR of **3m**

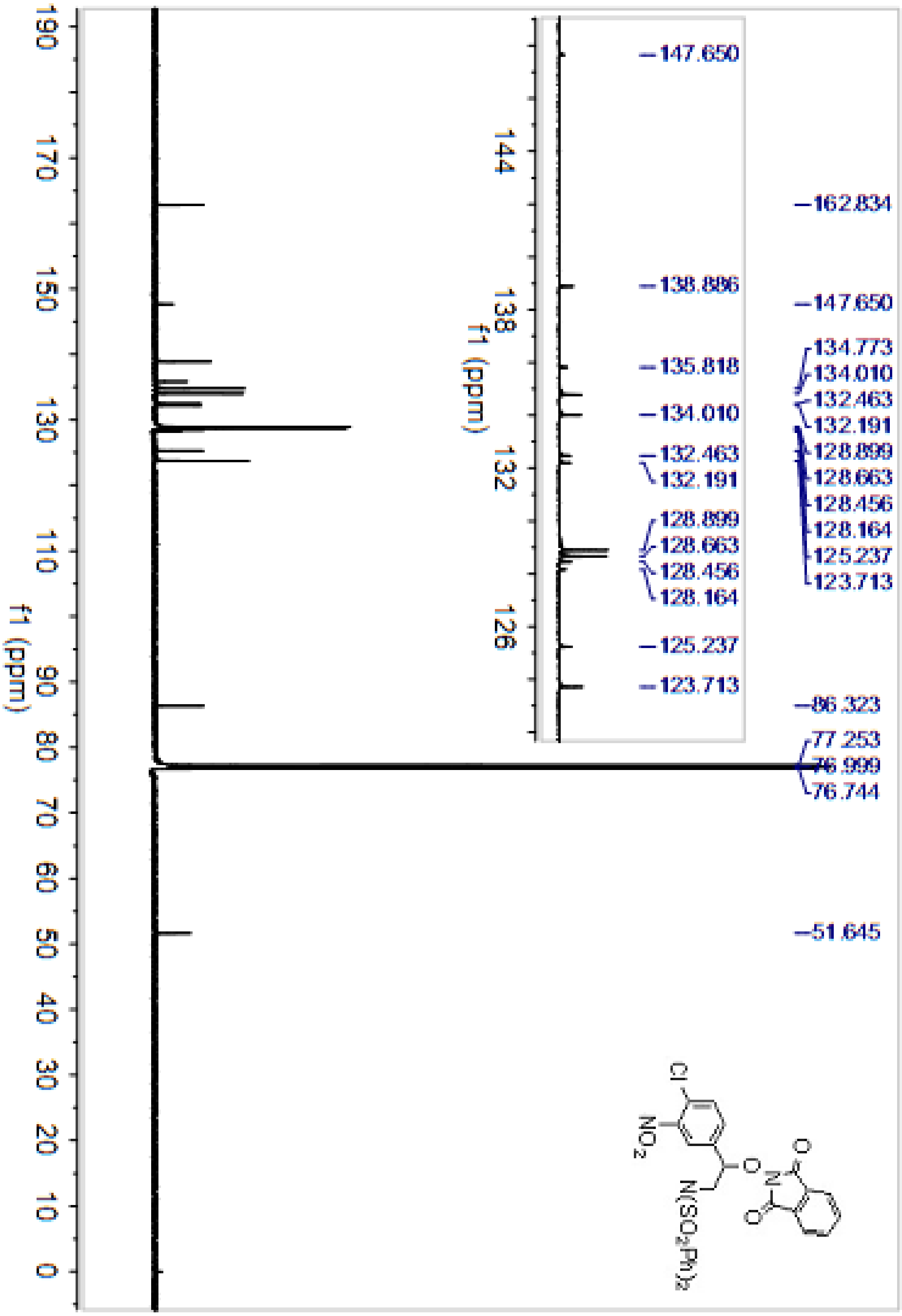

162.834
147.650
134.773
134.010
132.463
132.191
128.899
128.663
128.456
128.164
125.237
123.713
86.323
77.253
76.999
76.744
51.645
147.650
138.886
135.818
134.010
132.463
132.191
128.899
128.663
128.456
128.164
125.237
123.713
f1 (ppm)
f1 (ppm)
Cl
NO2
N(SO2Ph)2

^{1}H NMR of **3n**

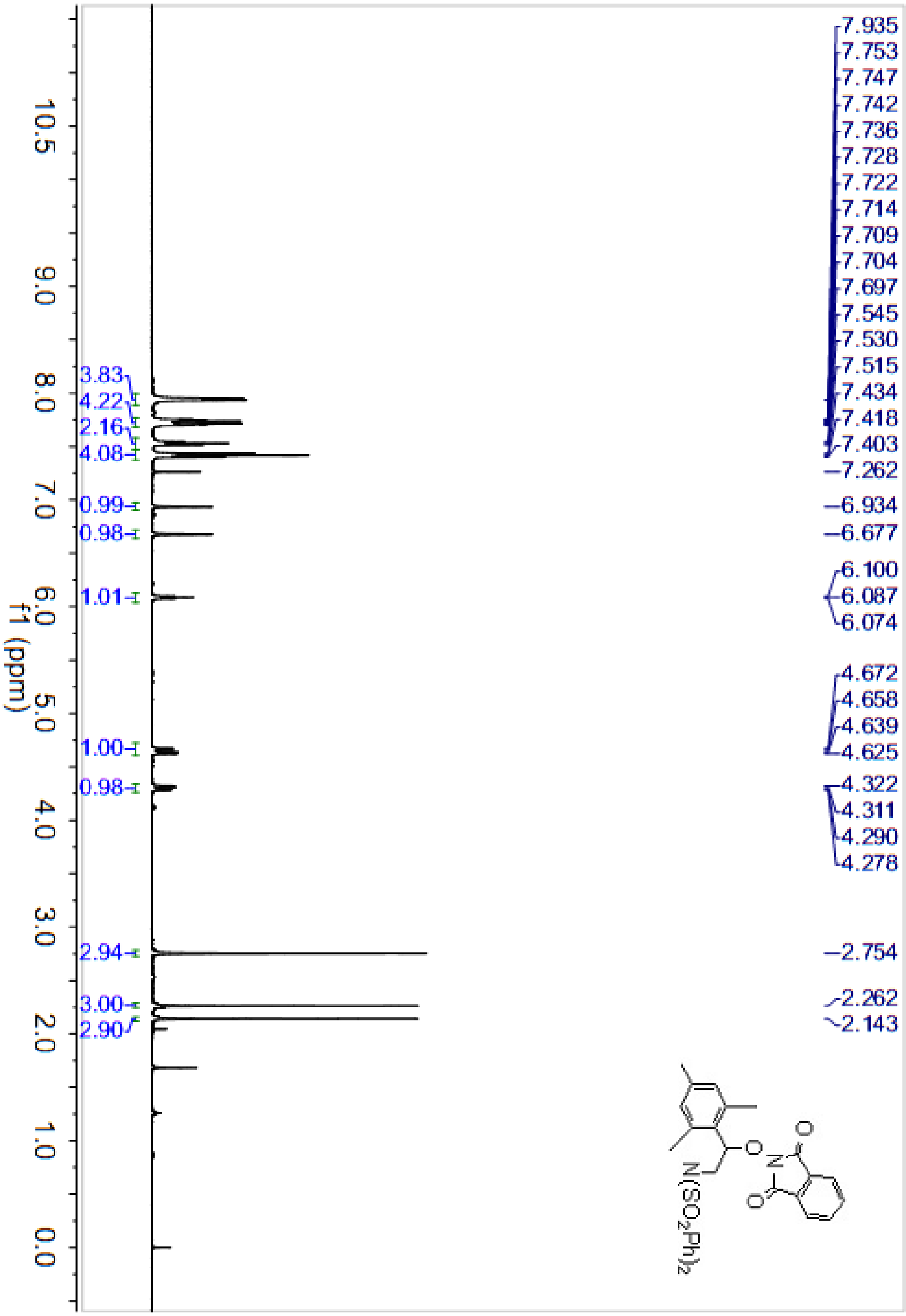
7.935
7.753
7.747
7.742
7.736
7.728
7.722
7.714
7.709
7.704
7.697
7.545
7.530
7.515
7.434
7.418
7.403
7.262
6.934
6.677
6.100
6.087
6.074
4.672
4.658
4.639
4.625
4.322
4.311
4.290
4.278
2.754
2.262
2.143
3.83
4.22
2.16
4.08
0.99
0.98
1.01
1.00
0.98
2.94
3.00
2.90
f1 (ppm)
N(SO2Ph)2

^{13}C NMR of **3n**

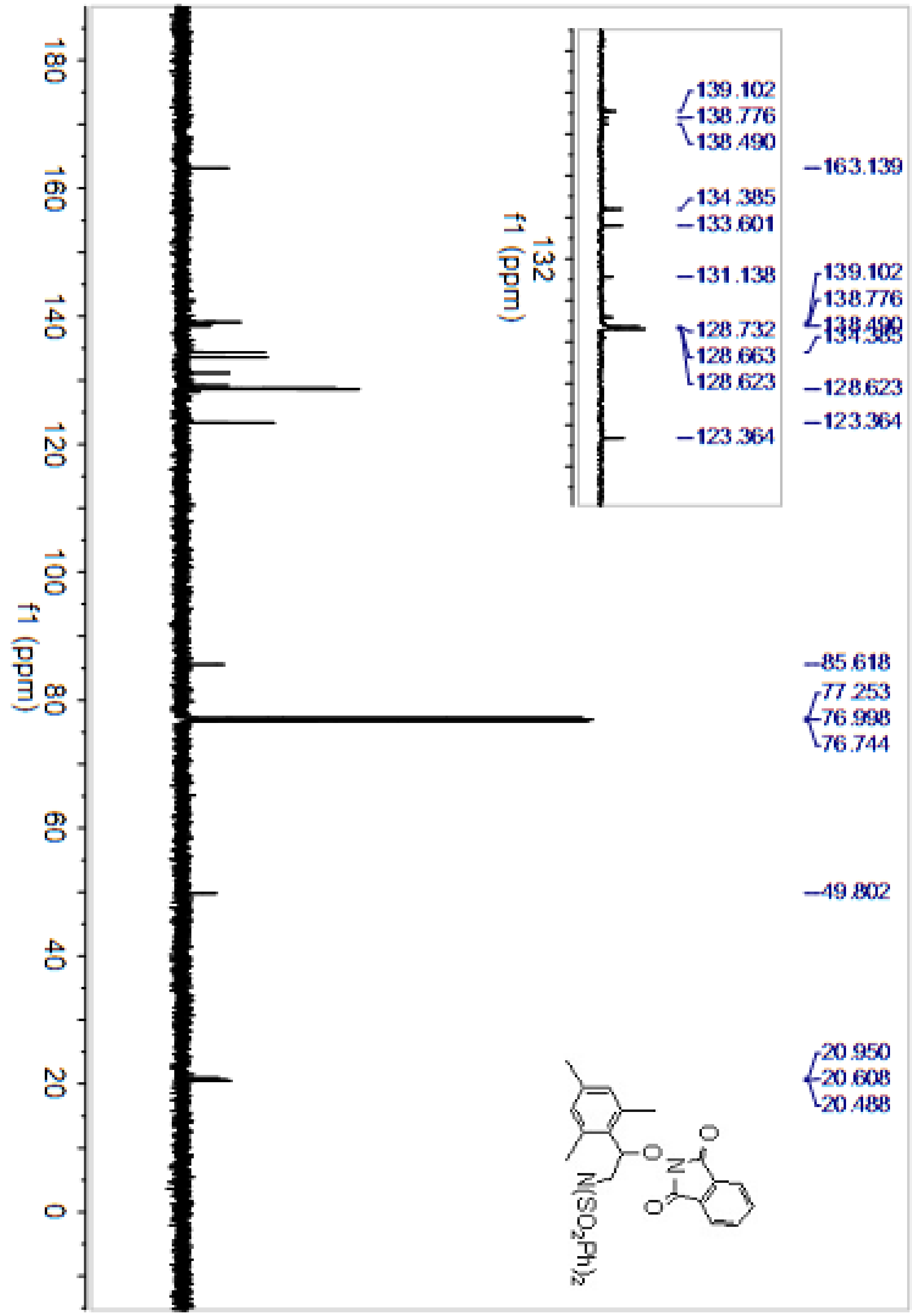
139.102
138.776
138.490
134.385
133.601
131.138
128.732
128.663
128.623
123.364
163.139
139.102
138.776
128.623
123.364
85.618
77.253
76.998
76.744
49.802
20.950
20.608
20.488
132
f1 (ppm)
180
160
140
120
100
80
60
40
20
0
f1 (ppm)

^{1}H NMR of **3o**

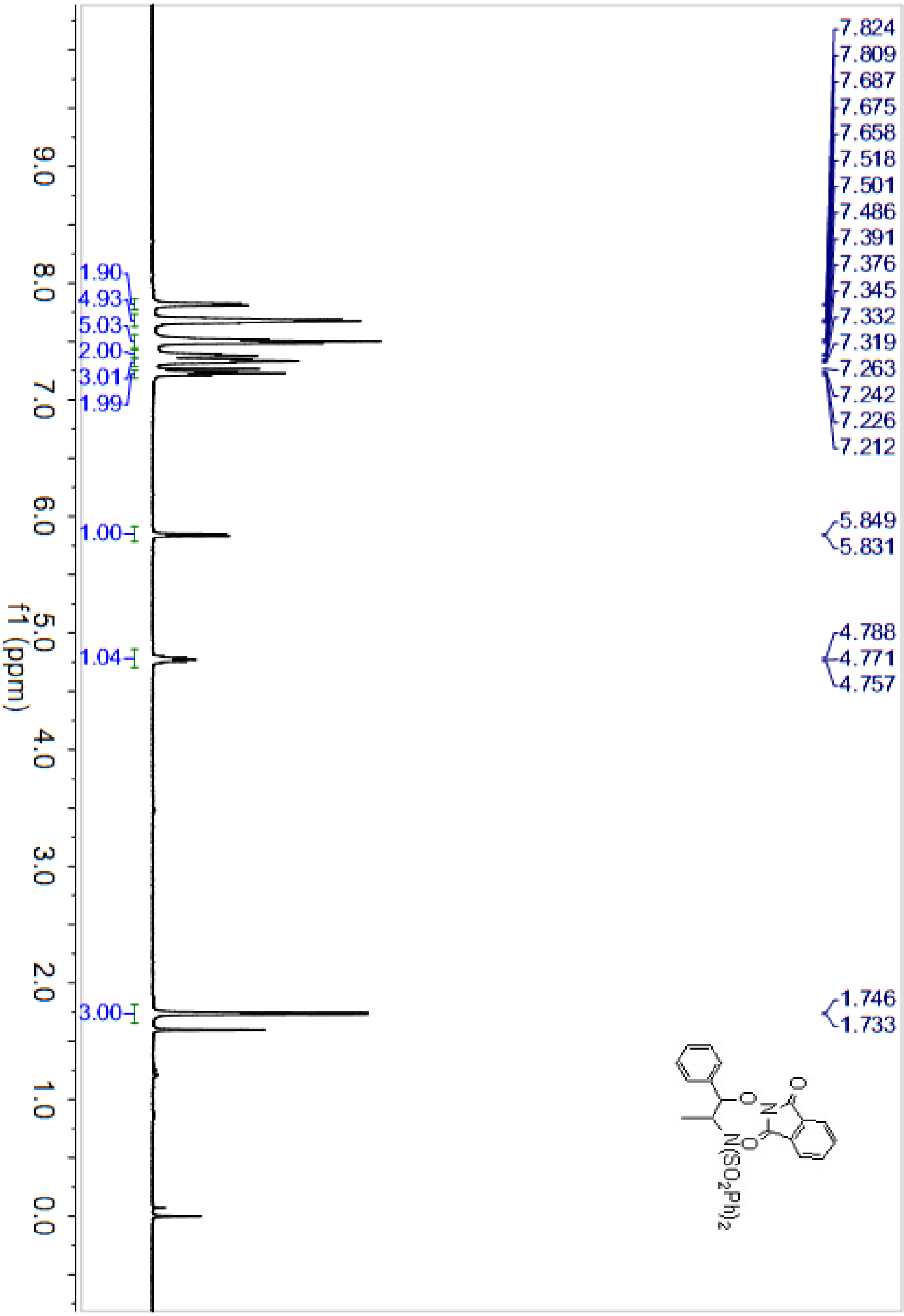
7.824
7.809
7.687
7.675
7.658
7.518
7.501
7.486
7.391
7.376
7.345
7.332
7.319
7.263
7.242
7.226
7.212
5.849
5.831
4.788
4.771
4.757
1.746
1.733
1.90
4.93
5.03
2.00
3.01
1.99
1.00
1.04
3.00
9.0
8.0
7.0
6.0
5.0
4.0
3.0
2.0
1.0
0.0
f1 (ppm)
N(SO2Ph)2

^{13}C NMR of **3o**

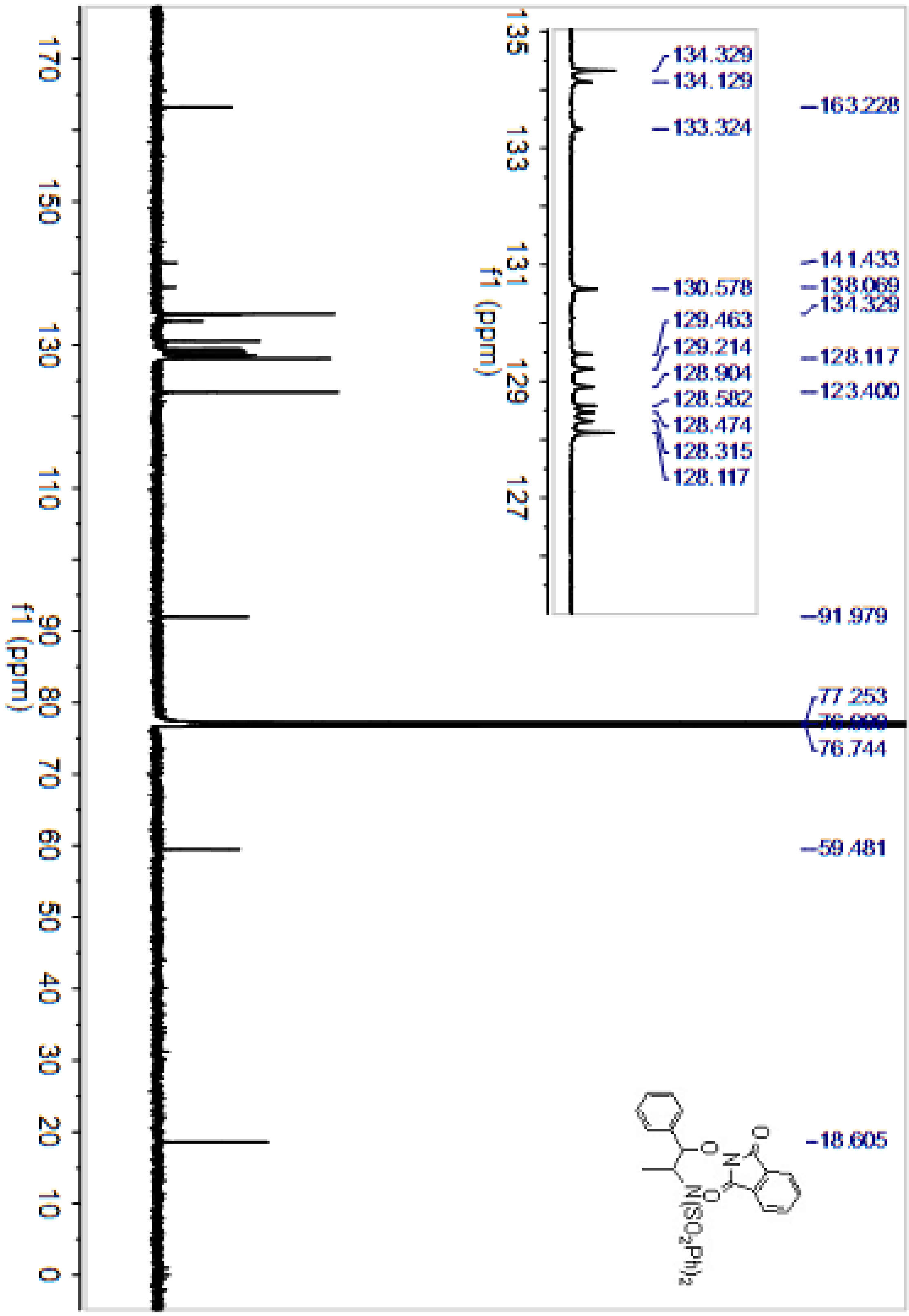
163.228
141.433
138.069
134.329
128.117
123.400
91.979
77.253
76.744
59.481
18.605
134.329
134.129
133.324
130.578
129.463
129.214
128.904
128.582
128.474
128.315
128.117
f1 (ppm)
N(SO2Ph)2

^{1}H NMR of **3p**

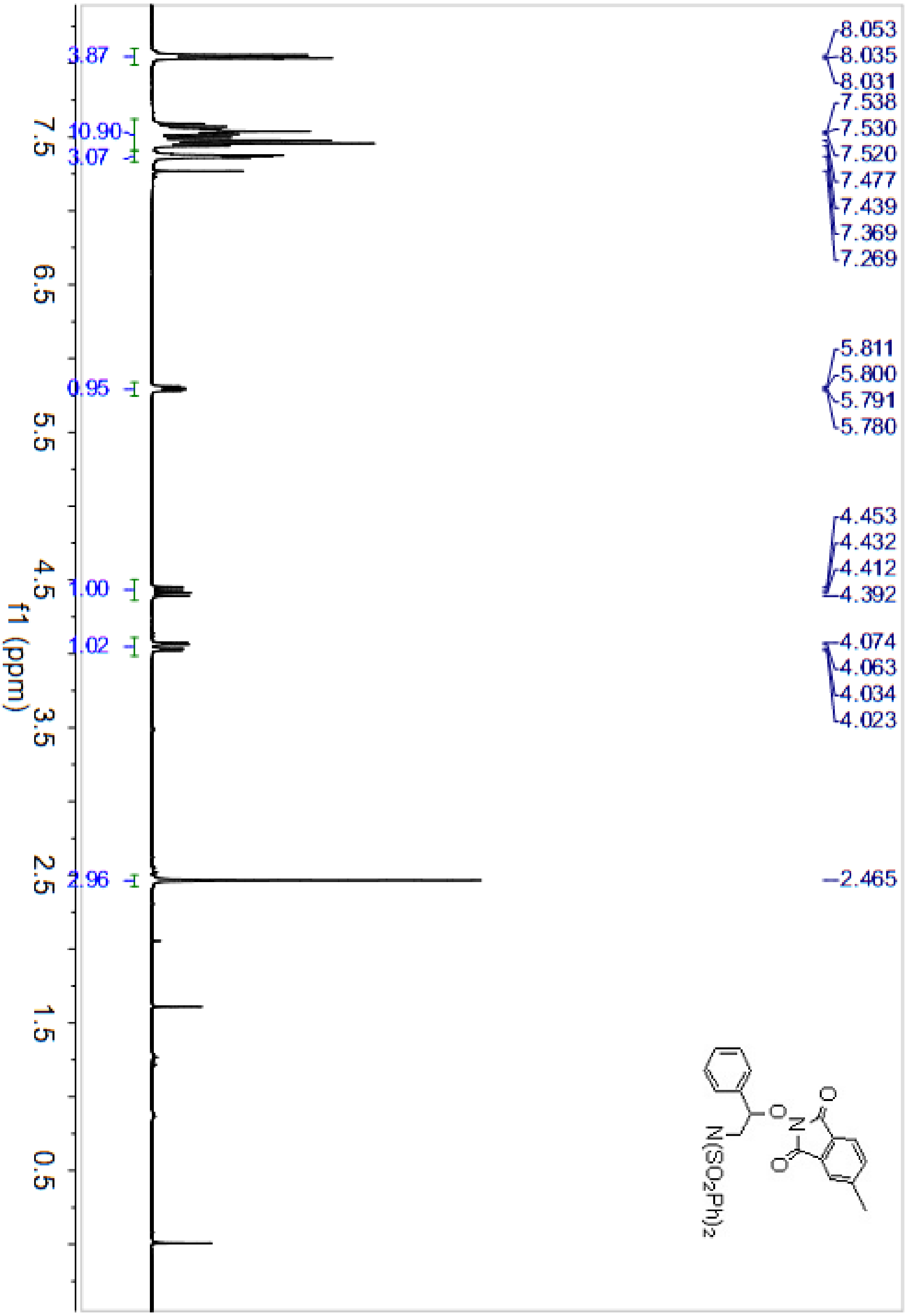

8.053
8.035
8.031
7.538
7.530
7.520
7.477
7.439
7.369
7.269
5.811
5.800
5.791
5.780
4.453
4.432
4.412
4.392
4.074
4.063
4.034
4.023
2.465
3.87
10.90
3.07
0.95
1.00
1.02
2.96
7.5
6.5
5.5
4.5
3.5
2.5
1.5
0.5
f1 (ppm)
N(SO2Ph)2

^{13}C NMR of **3p**

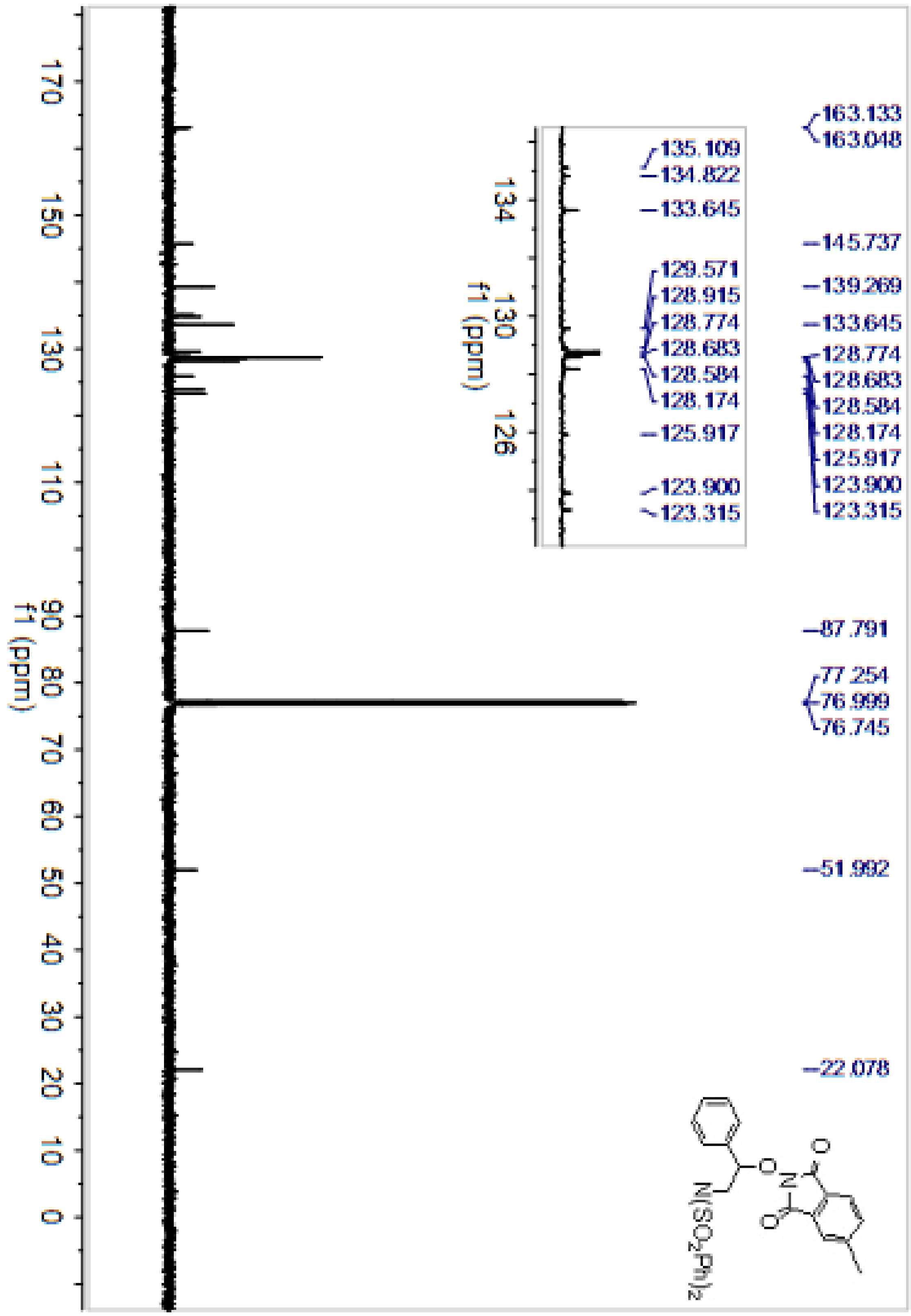
163.133
163.048
145.737
139.269
133.645
128.774
128.683
128.584
128.174
125.917
123.900
123.315
87.791
77.254
76.999
76.745
51.992
22.078
135.109
134.822
133.645
129.571
128.915
128.774
128.683
128.584
128.174
125.917
123.900
123.315
f1 (ppm)
N(SO2Ph)2

^{1}H NMR of **3q**

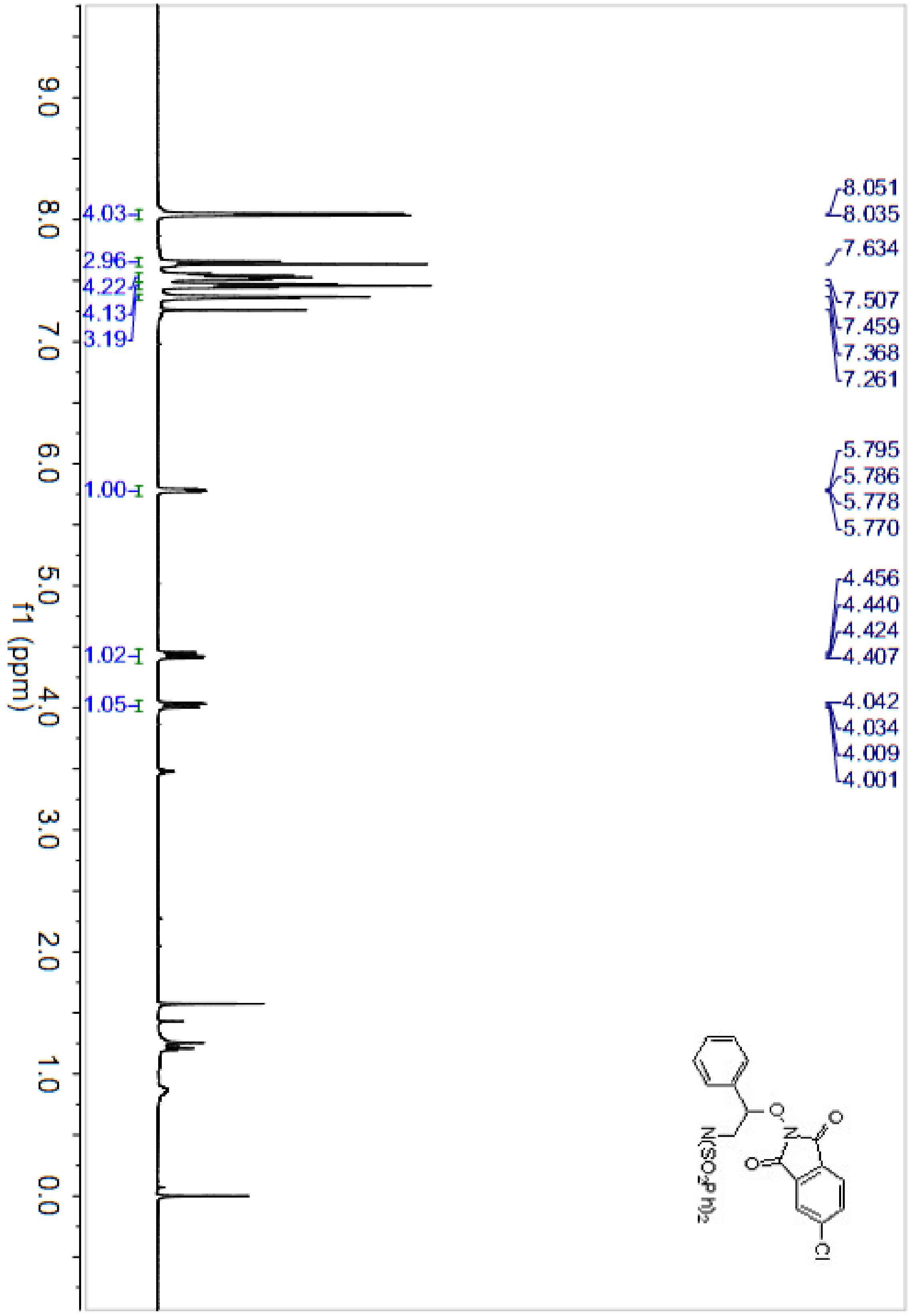
9.0
8.0
7.0
6.0
5.0
4.0
3.0
2.0
1.0
0.0
f1 (ppm)
4.03
2.96
4.22
4.13
3.19
1.00
1.02
1.05
8.051
8.035
7.634
7.507
7.459
7.368
7.261
5.795
5.786
5.778
5.770
4.456
4.440
4.424
4.407
4.042
4.034
4.009
4.001
N(SO2Ph)2
Cl

^{13}C NMR of **3q**

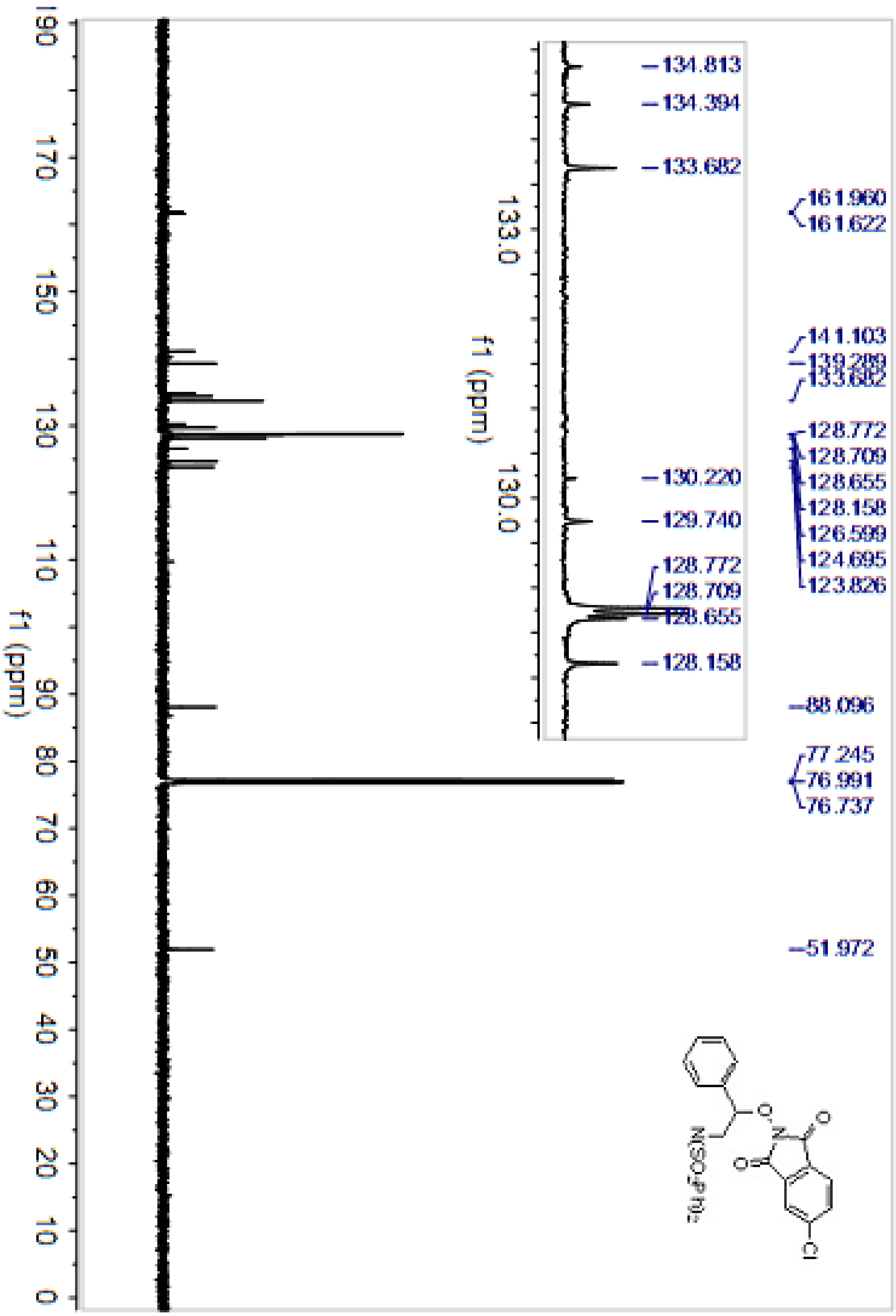

161.960
161.622
141.103
139.289
133.682
128.772
128.709
128.655
128.158
126.599
124.695
123.826
88.096
77.245
76.991
76.737
51.972
134.813
134.394
133.682
130.220
129.740
128.772
128.709
128.655
128.158
133.0
130.0
f1 (ppm)
190
170
150
130
110
90
80
70
60
50
40
30
20
10
0
f1 (ppm)

^{1}H NMR of **4**

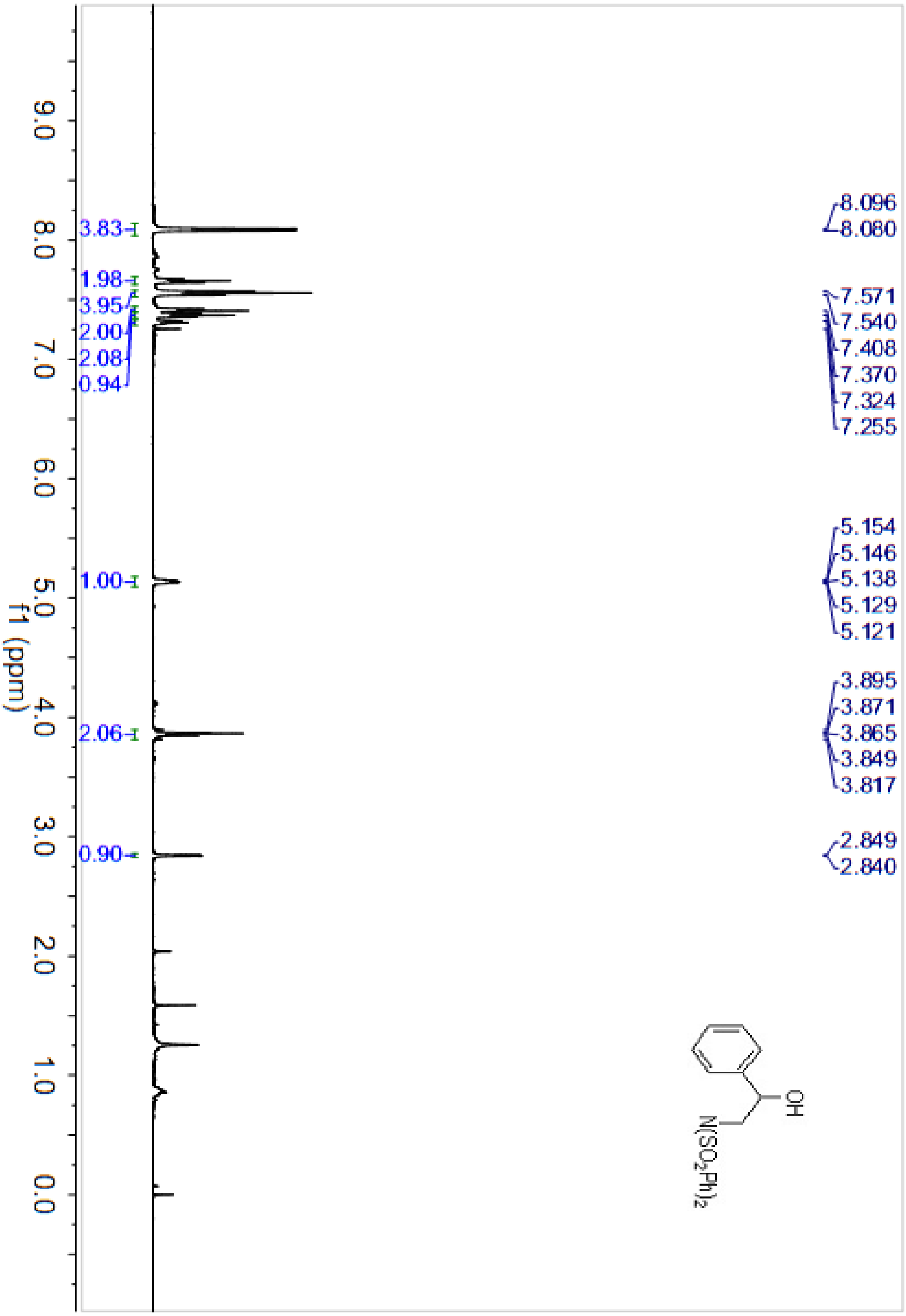
8.096
8.080
7.571
7.540
7.408
7.370
7.324
7.255
5.154
5.146
5.138
5.129
5.121
3.895
3.871
3.865
3.849
3.817
2.849
2.840
3.83
1.98
3.95
2.00
2.08
0.94
1.00
2.06
0.90
9.0
8.0
7.0
6.0
5.0
4.0
3.0
2.0
1.0
0.0
f1 (ppm)
OH
N(SO2Ph)2

^{13}C NMR of **4**

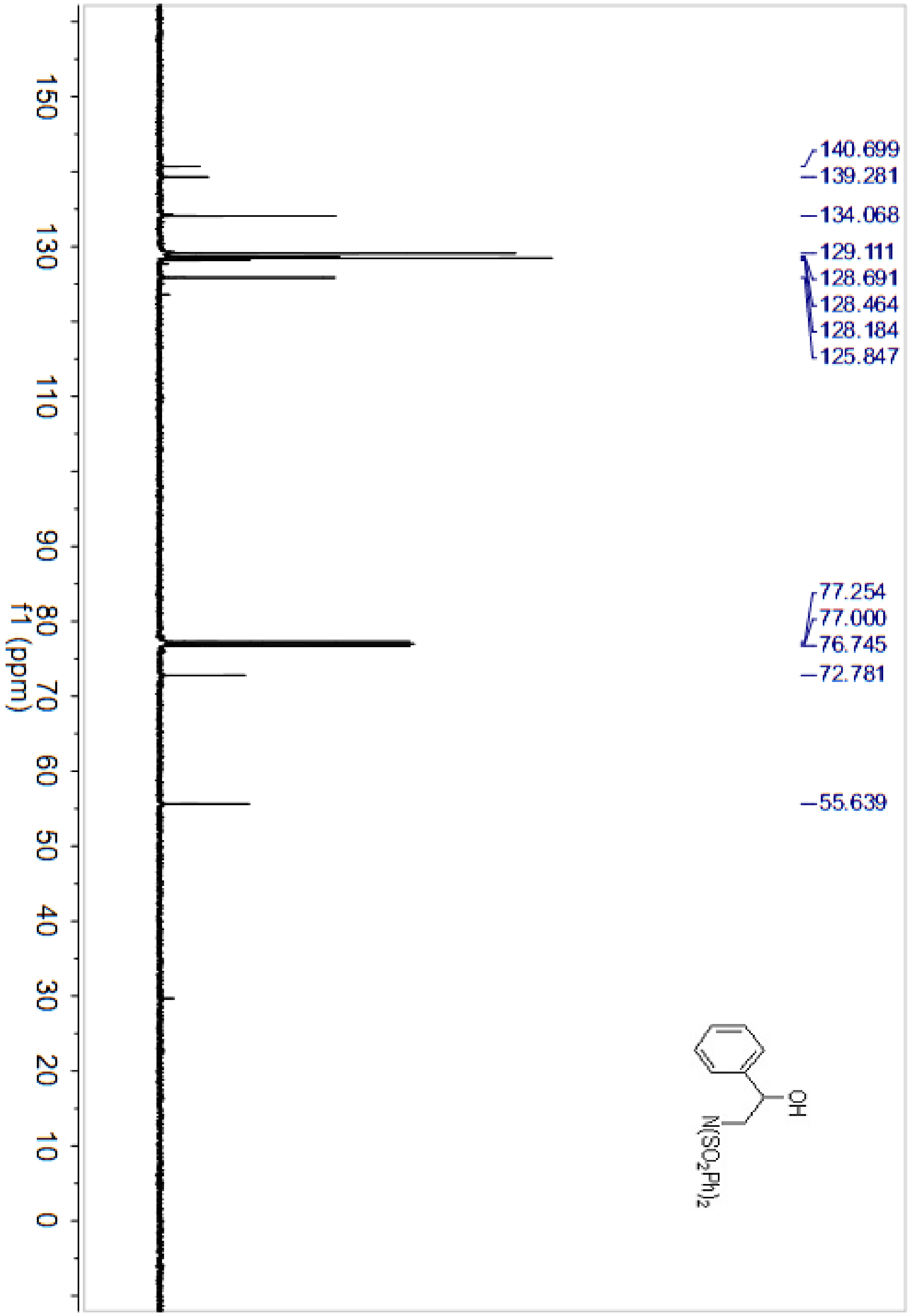
140.699
139.281
134.068
129.111
128.691
128.464
128.184
125.847
77.254
77.000
76.745
72.781
55.639
f1 (ppm)
150
130
110
90
80
70
60
50
40
30
20
10
0
OH
N(SO2Ph)2

^{1}H NMR of **5**

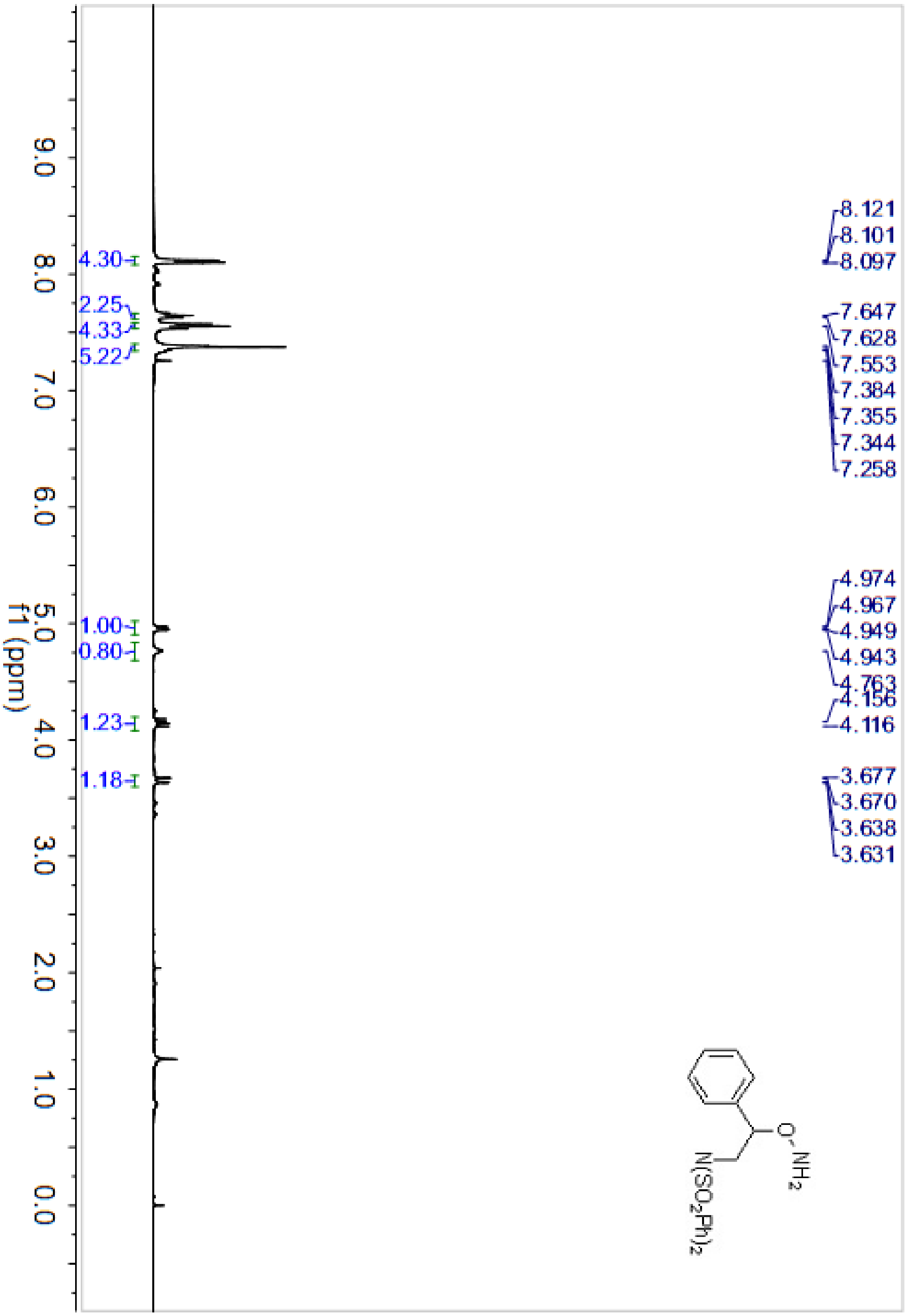
8.121
8.101
8.097
7.647
7.628
7.553
7.384
7.355
7.344
7.258
4.974
4.967
4.949
4.943
4.763
4.156
4.116
3.677
3.670
3.638
3.631
4.30
2.25
4.33
5.22
1.00
0.80
1.23
1.18
f1 (ppm)
O-NH2
N(SO2Ph)2

^{13}C NMR of **5**

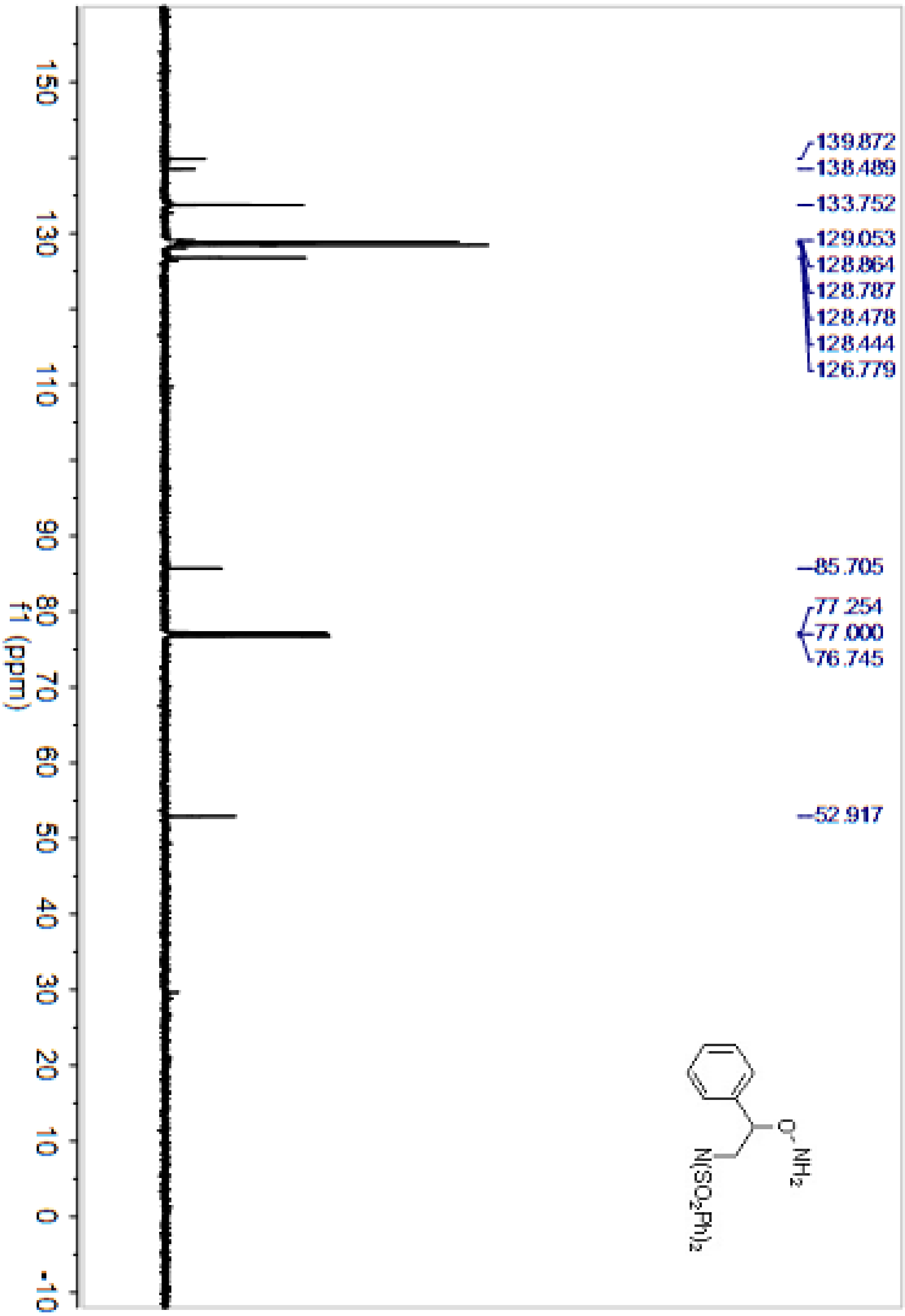
139.872
138.489
133.752
129.053
128.864
128.787
128.478
128.444
126.779
85.705
77.254
77.000
76.745
52.917
f1 (ppm)
150
130
110
90
80
70
60
50
40
30
20
10
0
-10
O NH2
N(SO2Ph)2
